# Impact of Surgery-Induced Myeloid-derived Suppressor Cells and the NOX2/ROS Axis on Postoperative Survival in Human Pancreatic Cancer

**DOI:** 10.1158/2767-9764.CRC-23-0447

**Published:** 2024-04-25

**Authors:** Hanna Grauers Wiktorin, Ebru Aydin, Roberta Kiffin, Caroline Vilhav, Johan Bourghardt Fagman, Mustafa Kaya, Sanchari Paul, Beatrice Westman, Svein Olav Bratlie, Peter Naredi, Kristoffer Hellstrand, Anna Martner

**Affiliations:** 1 TIMM Laboratory, Sahlgrenska Center for Cancer Research, Department of Microbiology and Immunology, Institute of Biomedicine, Sahlgrenska Academy, University of Gothenburg, Gothenburg, Sweden.; 2 Department of Molecular Genetics, German Cancer Research Center (DKFZ), Heidelberg, Germany.; 3 Department of Surgery, Institute of Clinical Sciences, Sahlgrenska Academy, University of Gothenburg, Gothenburg, Sweden.; 4 Department of Surgery, Sahlgrenska University Hospital, Gothenburg, Sweden.; 5 TIMM Laboratory, Sahlgrenska Center for Cancer Research, Department of Infectious Diseases, Institute of Biomedicine, Sahlgrenska Academy, University of Gothenburg, Gothenburg, Sweden.

## Abstract

Preclinical studies imply that surgery triggers inflammation that may entail tumor outgrowth and metastasis. The potential impact of surgery-induced inflammation in human pancreatic cancer is insufficiently explored. This study included 17 patients with periampullary cancer [pancreatic ductal adenocarcinoma (PDAC) *n* = 14, ampullary carcinoma *n* = 2, cholangiocarcinoma *n* = 1] undergoing major pancreatic cancer surgery with curative intent. We analyzed the potential impact of preoperative and postoperative immune phenotypes and function on postoperative survival with >30 months follow-up. The surgery entailed prompt expansion of monocytic myeloid-derived suppressor cells (M-MDSC) that generated NOX2-derived reactive oxygen species (ROS). Strong induction of immunosuppressive M-MDSC after surgery predicted poor postoperative survival and coincided with reduced functionality of circulating natural killer (NK) cells. The negative impact of surgery-induced M-MDSC on survival remained significant in separate analysis of patients with PDAC. M-MDSC–like cells isolated from patients after surgery significantly suppressed NK cell function *ex vivo*, which was reversed by inhibition of NOX2-derived ROS. High NOX2 subunit expression within resected tumors from patients with PDAC correlated with poor survival whereas high expression of markers of cytotoxic cells associated with longer survival. The surgery-induced myeloid inflammation was recapitulated *in vivo* in a murine model of NK cell–dependent metastasis. Surgical stress thus induced systemic accumulation of M-MDSC–like cells and promoted metastasis of NK cell–sensitive tumor cells. Genetic or pharmacologic suppression of NOX2 reduced surgery-induced inflammation and distant metastasis in this model. We propose that NOX2-derived ROS generated by surgery-induced M-MDSC may be targeted for improved outcome after pancreatic cancer surgery.

**Significance:**

Pancreatic cancer surgery triggered pronounced accumulation of NOX2^+^ myeloid-derived suppressor cells that inhibited NK cell function and negatively prognosticated postoperative patient survival. We propose the targeting of M-MDSC as a conceivable strategy to reduce postoperative immunosuppression in pancreatic cancer.

## Introduction

Cancer in the pancreas and adjacent tissues is a major cause of cancer-related death worldwide (1). Neoplasms occurring in vicinity of the ampulla of Vater are termed periampullary cancer where the by far most common variant is pancreatic ductal adenocarcinoma (PDAC; ref. 2). Approximately 80% of patients with periampullary cancer present with locally advanced disease or distant metastasis (2–4) and are not candidates for surgery with curative intent. In patients with resectable disease, the use of adjuvant or neoadjuvant multiagent chemotherapy has improved long-term outcomes (5, 6). Despite these advances, many patients develop local recurrence or distant metastasis with poor prospects for long-term survival (7, 8) thus highlighting the need for novel or supplementary therapies that may reduce the risk of postoperative metastasis.

Myeloid-derived suppressor cells (MDSC) are immature immunosuppressive myeloid cells that accumulate in several forms of cancer (9). MDSC, which often indicate advanced disease, utilize multiple mechanisms of immunosuppression including the formation and extracellular release of reactive oxygen species (ROS) generated by the NOX2 enzyme. These toxic oxygen derivatives suppress several aspects of antitumor immunity, including natural killer (NK) cells and cytotoxic T cells (9–11). Human monocytic MDSC (M-MDSC) are phenotypically defined by expression of CD14 in conjunction with low-grade expression of HLA-DR whereas granulocytic MDSC (G-MDSC) carry the CD11b^+^CD15^+^HLA-DR^−^ phenotype (12). In experimental pancreatic cancer, MDSC were reported to contribute to immunosuppression (13, 14) and in patients with pancreatic cancer, high levels of MDSC were suggested to limit the efficacy of cytokine-induced killer cell immunotherapy or chemotherapy (15, 16).

Earlier studies of the tumor microenvironment of human pancreatic cancer reveal complex immune escape mechanisms, which likely derive from chronic inflammation comprising a high density of infiltrating immunosuppressive myeloid cells and scarce presence of potentially antineoplastic NK or T cells (17–21). These characteristics are, along with a low mutational burden of tumor cells, a desmoplastic stroma and a poorly developed microvasculature, proposed to explain why immunotherapies, including antibodies that target CTLA-4 or the PD-1–PD-L1 interaction, have not yielded practice-changing results in pancreatic cancer (17, 22). The chronic inflammation of pancreatic cancer tumors is accompanied by systemic inflammation with accumulation of myeloid cells in blood. Whereas high preoperative inflammation scores reportedly associate with poor survival in periampullary cancer (23–25), few studies have addressed how pancreatic cancer surgery influences inflammation and MDSC or its potential prognostic impact.

Surgery is the only curative option in periampullary cancer. Pancreaticoduodenectomy (Whipple procedure) encompasses removal of the pancreas head, the gall bladder and bile duct, part of the stomach, duodenum, proximal jejunum, and adjacent lymph nodes (26). If tumors are present also in the tail region, the surgery comprises complete pancreatectomy. For this study, we examined whether the magnitude of systemic inflammation triggered by these major surgical procedures might influence immunoreactivity. We report that surgery entails systemic expansion of immunosuppressive M-MDSC that independently predicted poor long-term survival. We also performed *ex vivo* experiments to decipher mechanisms of relevance to surgery-induced immunosuppression with focus on the interaction between NK cells and M-MDSC–derived ROS produced by the NOX2 enzyme of myeloid cells. Finally, we examined the potential role of surgical stress on metastasis formation in a NK cell–dependent tumor model *in vivo* (27). Our findings imply that M-MDSC may be targeted for improved perioperative immunosurveillance in patients undergoing surgery for periampullary cancer.

## Materials and Methods

### Immune Phenotype Pancreatic Cancer Study and Patient Characteristics

**FIGURE 1:**
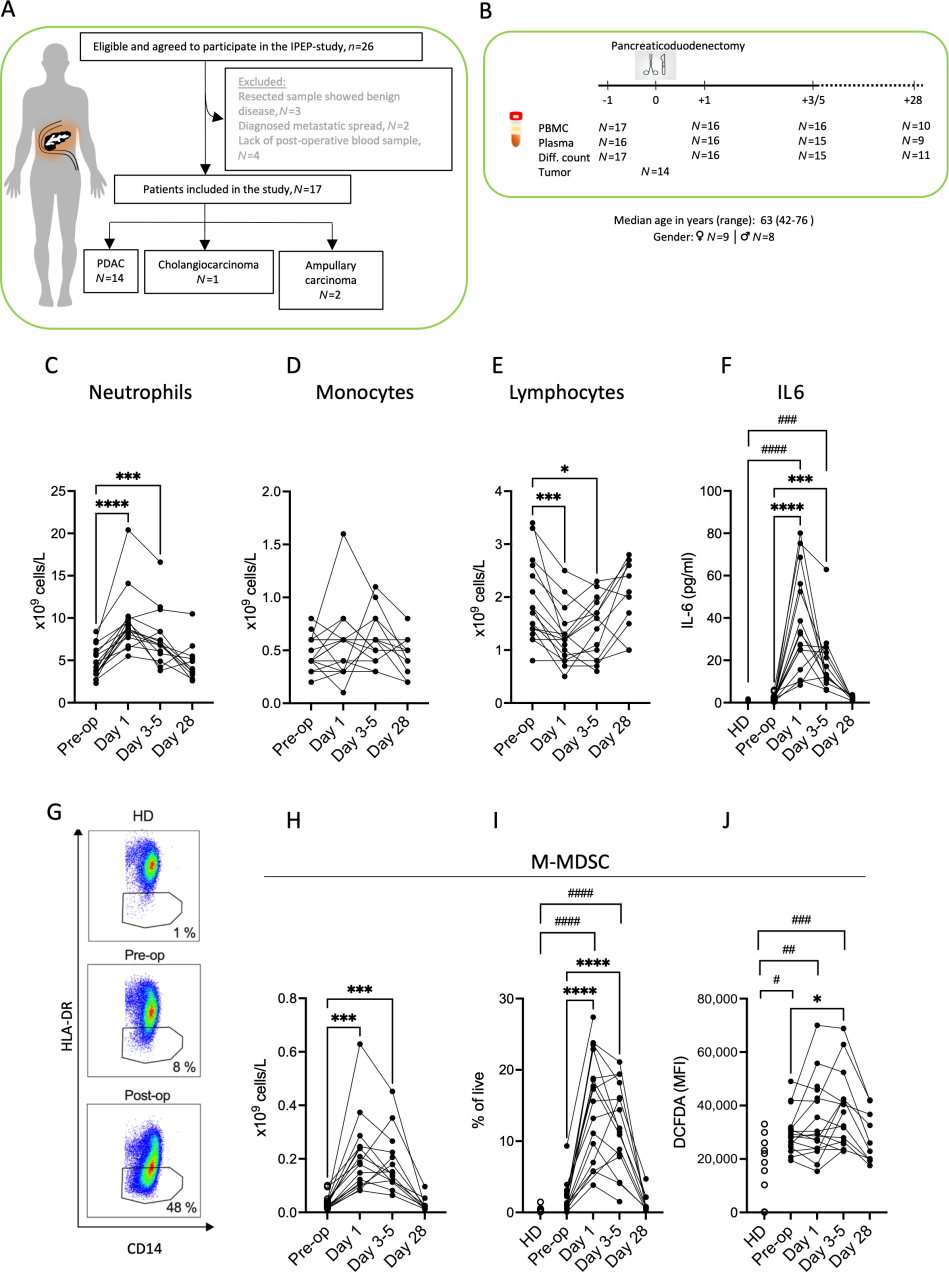
Pancreatic cancer surgery triggers inflammation and expansion of M-MDSC. **A,** Flow chart of included IPEP study participants. **B,** Study design and available samples. Absolute numbers of neutrophils (**C**), monocytes (**D**), and lymphocytes (**E**) in patients undergoing surgery for periampullary cancer with whole blood samples (differential counts) analyzed before (pre-op) and 1, 3–5 and 28 days after pancreatic cancer surgery (day 1, days 3–5, and day 28, respectively; *N* = 17 for pre-op, *N* = 16 for day 1, *N* = 15 for days 3–5, *N* = 11 for day 28). **F,** Plasma levels of IL6 in healthy donors (HD) and in pre- and post-op samples from patients (*N* = 16 for pre-op and for day 1, *N* = 15 for days 3–5, *N* = 9 for day 28, *N* = 6 for HD). **G–I,** CD14^+^HLADR^low^ M-MDSC in peripheral blood of healthy donors and patients before and after surgery analyzed by flow cytometry. **G,** Representative FACS plot of the frequency of CD14^+^HLADR^low^ cells among CD14^+^ cells. Absolute numbers (**H**) and frequencies (**I**) of M-MDSC (*N* = 17 for pre-op, *N* = 16 for day 1 and days 3–5, *N* = 10 for day 28, *N* = 12 for HD). **J,** M-MDSC levels of ROS as measured by DCFDA MFI (*N* = 16 for pre-op, for day 1 and for days 3–5, *N* = 10 for day 28, *N* = 12 for HD). Preoperative and postoperative samples were compared using mixed-effects analysis followed by Šídák multiple comparison test (*P*-values indicated by *). HD and cancer samples were compared using Kruskal–Wallis test followed by Dunn multiple comparison test (*P*-values indicated by ^#^). *^/#^*P* < 0.05, ^##^*P* < 0.01, ***^/###^*P* < 0.001, ****^/####^*P* < 0.0001.

Study patients were included in the immune phenotype pancreatic cancer (IPEP) trial, ethical approval number 057-18 (approved March 7, 2018, Regional Ethics Review Board in Gothenburg). The IPEP trial aims to define the impact of pancreaticoduodenectomy on the phenotype and function of immune cells along with its influence on immune parameters and disease outcome. Twenty-six patients eligible for pancreaticoduodenectomy with suspected periampullary cancer were enrolled between September 2018 and November 2019; 3 patients were excluded from survival analysis because resected sample showed benign disease; 2 patients were excluded after diagnosis of metastatic spread during surgery; and 4 patients were excluded because of the absence of postoperative blood samples. In two cases, tumors were detected also in the tail region, and complete pancreatectomy was thus performed. Of the 17 included patients with confirmed periampullary cancer, 14 were diagnosed with PDAC, 2 with ampullary carcinoma and 1 with cholangiocarcinoma (Fig. 1A). The median age was 63 years (range: 42–76) and 9 (53%) were female. Table 1 accounts for additional patient characteristics along with the impact of these parameters on postoperative survival. The study was conducted in accordance with the Declaration of Helsinki. All patients gave written informed consent before enrolment. Ten patients died during the study period.

**TABLE 1:** Patient characteristics and their impact on survival

Characteristics		Number of patients (%)	Survival months, mean (range)	HR (95%CI)	*P*-value
Age (years), median (range)		63 (42–76)		1.07 (0.96–1.19)	0.21
Gender	Male	9 (53)	25.1 (6.7–45.6)	0.80 (0.23–2.77)	0.72
	Female	8 (47)	29.5 (5.6–46.6)		
TNM Classification^a^:	T1	2 (12)	36.3 (35.3–37.2)	1.57 (0.56–4.47)	0.39
Tumor stage	T2	10 (59)	29.1 (11.4–46.6)		
	T3	5 (29)	20.7 (5.6–45.6)		
TNM Classification^a^:	N0	3 (18)	34.4 (11.0–46.6)	2.2 (0.81–5.93)	0.12
Lymph node stage	N1	7 (41)	28.9 (14–45.6)		
	N2	7 (41)	23.0 (5.6–37.2)		
Pathologic diagnosis	PDAC^b^	14 (82)	29.2 (11.0–46.6)	3.11 (1.04–9.29)	0.042
	CC^c^	1 (6)	45.6		
	AC^d^	2 (12)	6.15 (5.6–6.7)		
Vascular resection	Vein	8 (47)	32.3 (14–46.6)	2.05 (0.57–7.32)	0.27
	No	9 (53)	23.2 (5.6–45.6)		
ASA classification^e^	1	5 (29)	35.1 (11.0–45.6)	1.50 (0.65–3.46)	0.34
	2	8 (47)	22.2 (5.6–46.6)		
	3	4 (24)	28.3 (13.4–35.3)		
Type of surgery	Pancreaticoduodenectomy	15 (88)	26.8 (5.6–46.6)	0.72 (0.089–5.8)	0.75
	Complete pancreatectomy	2 (12)	32.6 (30.7–34.4)		
CD^f^ Classification of complications	0	3 (18)	19.0 (6.7–36.2)	1.03 (0.48–2.2)	0.95
	1	3 (18)	41.7 (34.0–45.6)		
	2	9 (53)	26.5 (5.6–46.6)		
	3	2 (12)	23.2 (11.0–35.3)		
Surgery duration (hours), median (range)		6.7 (5.5–10.4)		1.00 (0.99–1.01)	0.93
Adjuvant chemotherapy	FOL^g^/GemCap^h^^,^^i^	9 (53)	30.0 (11.0–45.6)	1.93 (0.73–5.12)	0.18
	Gemcitabine	4 (35)	35.1 (14.0–46.6)		
	No	2 (12)	18.7 (6.7–30.7)		
	Unknown	2 (12)	9.5 (5.6–13.4)		

NOTE: Statistics by Cox regression.

^a^Union for International Cancer Control (UICC) TNM Classification of malignant tumors, 8th edition. Tumor (T), Lymph nodes (N), Metastases (M).

^b^Pancreatic ductal adenocarcinoma.

^c^Cholangiocarcinoma.

^d^Ampullary carcinoma.

^e^American Society of Anaesthesiologists physical status classification.

^f^Clavien-Dindo.

^g^FOLFORINOX.

^h^Gemcitabine + Capecitabine.

^i^One patient received neoadjuvant therapy in addition to adjuvant therapy.

### Isolation of Peripheral Blood Mononuclear Cells and Plasma

Peripheral venous blood samples for differential counts, isolation of mononuclear cells and plasma were collected from patients the day before and 1, 3–5, and 28 days after pancreaticoduodenectomy. Figure 1B accounts for the number of samples obtained for the different analyses. The blood was collected in BD Vacutainer CPT Sodium Citrate/Ficoll tubes. After centrifugation at 1,500 RCF for 20 minutes, plasma was collected and frozen. The peripheral blood mononuclear cell (PBMC) layer was washed twice with RPMI medium and cryopreserved. PBMC were additionally isolated from buffy coats from anonymized healthy donors by density centrifugation followed by two washes and cryopreservation. Anonymized plasma from healthy donors was obtained from the Sahlgrenska Blood Center, Gothenburg, Sweden.

### RNA Extraction and qRT-PCR

Snap frozen pancreatic cancer tumor biopsies were available from 14 of the included patients. RNA was extracted using RNeasy Plus Mini Kit (Qiagen GmbH) and quantified using NanoDrop. Reverse transcription was carried out using TATAA GrandScript cDNA Synthesis Kit (TATAA Biocenter). Quantitative measurements of *NCF2*, *CYBB, CD68*, *CD163,* and *NCAM1* expression were performed using TATAA SYBR GrandMaster Mix (TATAA Biocenter) in a Bio-Rad CFX 384 real-Time PCR system, following the manufacturer's recommendations. The expression was related to expression of the house keeping genes *RPS26* and *RPL7*, and relative expression was defined by 2^−ΔCt^. The primer sequences are listed in Supplementary Table S1.

### Cytokine Array Assay

Plasma from patients and healthy donors was analyzed using a 27-plex cytokine panel (BIO-Plex Pro Human Cytokine Standard 27-Plex Group 1, Bio-Rad, #M500KCAF0Y) using Luminex technology according to the manufacturer's instructions. In brief, magnetic antibody-coated beads were added to each well of the assay plates. After washing, 1:4 diluted plasma samples were added and plates were incubated for 30 minutes, washed, and incubated for another 30 minutes with detection antibodies. After washing, the plate was incubated with streptavidin-phycoerythrin for 10 minutes and cytokine levels were estimated using a Bio-Plex 200 System (Bio-Rad).

### Immunophenotyping of PBMC

Viably cryopreserved PBMC were thawed and stained with LIVE/DEAD Fixable yellow Dead stain kit (Thermo Fisher Scientific) for a myeloid and T-cell panel or with LIVE/DEAD fixable violet Dead stain kit (Thermo Fisher Scientific) for an NK cell panel for 20 minutes at 4°C. Cells were thereafter washed and stained with a myeloid panel of antibodies comprising CD1c-BV412 (331526, BioLegend), CD11b-PE (555388, BD Biosciences), CD14-APC-Cy7 (333951, BD Biosciences), CD141-APC (564123, BD Biosciences), CD16-BV605 (563172, BD Biosciences), CD33-PE-Cy7 (333952, BD Biosciences), CD86-BV711 (563158, BD Biosciences), HLA-ABC-BV605 (311432, BD BioLegend), HLA-DR-BV786 (564041, BD Biosciences), PD-L1-BV395 (740320, BD Biosciences), CD3-PerCp-Cy5.5 (332771, BD Biosciences), CD19-PerCp-Cy5.5 (332780, BD Biosciences), an NK cell panel of antibodies comprising CD3-BV510, CD19-BV510 (562947, BD Biosciences), CD14-BV510 (563079, BD Biosciences), CD56-BV786 (564058, BD Biosciences), CD16-APC-Cy7 (557758, BD Biosciences), CD57-FITC (333169, BD Biosciences), DNAM-1 (338314, BioLegend), NKG2D-PE-Cy7 (562365, BD Biosciences), NKp46-APC (130-092-609, Miltenyi Biotech), NKp30-BV711 (563383, BD Biosciences), NKG2A-PE (IM3291U, Beckman Coulter Diagnostics), and a T-cell panel of antibodies comprising CD3-BV711 (563725, BD Biosciences), CD4-APC-H7 (560158, BD Biosciences), CD8-PerCp-Cy5.5 (560662, BD Biosciences), CD45RA-APC (550855, BD Biosciences), CD45-RO-PE (554993, BD Biosciences), CCR7-PE-Cy7 (353226, BioLegend), CD25-AlexaFlur700 (561398, BD Biosciences), CD127- BV395 (742547, BD Biosciences), HLA-DR-FITC (347300, BD Biosciences), and PD-1-BV421 (562516, BD Biosciences). Cells were incubated with antibodies for 30 minutes at 4°C, washed and acquired on a five laser BD LSR Fortessa and analyzed with FlowJo (version 10 or later, BD Biosciences).

### ROS Measurement by DCFDA

The viably cryopreserved PBMC were thawed and stained with LIVE/DEAD fixable violet Dead Stain Kit (Thermo Fisher Scientific) for 20 minutes followed by staining with a myeloid panel of antibodies comprising CD14-APC-Cy7 (564123), CD33-PE-Cy7 (333952), CD16-BV605 (563172), HLA-DR-BV786 (564041), all from BD Biosciences, for 30 minutes at 4°C. Cells were then washed and stained with H2-DCFDA (D399, Molecular Probes) in serum-free Iscoves’ Modified Dulbecco's Medium (IMDM; 36531, Thermo Fisher Scientific) for 30 minutes at 37°C, washed and acquired on a five laser BD LSR Fortessa and analyzed with FlowJo.

### Culture of K562 and PANC-1 Target Cells in Microcytotoxicity Assays

K562 erythroleukemic cells (CCL-2243, ATCC), a prototypic NK cell target, were cultured in IMDM supplemented with 10% heat-inactivated FCS (PAA Laboratories GmbH), 100 µg/mL penicillin (Sigma), 100 µg/mL streptomycin (Sigma), 1 mmol/L sodium pyruvate (Gibco), and 2 mmol/L l-glutamine (Gibco) at 37°C, 5% CO_2_. Cell line authentication and *Mycoplasma* testing (Eurofins Genomics) were performed in 2022 and cells were used within approximately 20 passages. PANC-1 cells (CLR-1469, ATCC) were purchased in 2020. Cell line authentication and *Mycoplasma* testing was performed by ATCC. Cells were cultured in DMEM (Gibco) supplemented with 10% heat-inactivated FCS (PAA Laboratories GmbH), 100 µg/mL penicillin (Sigma), 100 µg/mL streptomycin (Sigma), 1 mmol/L sodium pyruvate (Gibco), and 4 mmol/L l-glutamine (Gibco) at 37°C, 5% CO_2_ and used within 20 passages.

### Isolation of PBMC and NK Cells from Healthy Donor Buffy Coats

PBMC were prepared from healthy donor blood buffy coats by Ficoll-Paque (Lymphoprep, 07861, Stemcell Technologies) density centrifugation. NK cells were isolated from PBMC by negative magnetic label separation using a human NK cell isolation kit (Miltenyi Biotec) according to manufacturer's instructions. The purity was consistently >90%. Isolated NK cells were cultured in round-bottom 96-well plates overnight in IMDM supplemented with 10% heat-inactivated FCS and 100 U/mL recombinant human IL2 (Proleukin, Eurocetus) with or without the addition of monocytes, NOX2 inhibitors or other compounds as described below.

### Isolation of CD33^+^ and Pan-monocytic Myeloid Cells from PBMC

CD33^+^ cells were isolated from cryopreserved PBMC samples obtained from healthy donor buffy coats or patient blood samples by positive magnetic selection using human anti-CD33 coated MicroBeads (130-045-501, Miltenyi Biotec) according to manufacturers’ instructions with a purity >90%. Pan-monocytes were isolated from freshly isolated PBMC by negative selection using magnetic microbead depletion according to the manufacturer's (Miltenyi Biotec) instructions with a monocyte purity of 60%–85%.

### Assay of Myeloid Cell–induced Suppression of NK Cell Function

The enriched NK cells from healthy donor PBMC were stimulated overnight with IL2 and then cocultured with K562 target cells and healthy donor or patient-derived preoperative or postoperative CD33^+^ cells at a 1:1:1 ratio in 96-well plates. Experiments were performed in the presence or absence of 100 µmol/L histamine dihydrochloride (HDC; Sigma-Aldrich) in IMDM supplemented with 10% heat-inactivated FCS and anti-CD107a-BUV395 (565113, BD Biosciences) for 4 hours at 37°C and 5% CO_2_. After incubation, cells were washed and stained with LIVE/DEAD fixable violet Dead stain kit (Thermo Fisher Scientific) and anti-CD16-AlexaFluor700 (557920, BD Biosciences), anti-CD56-BV711 (563169, BD Biosciences), and CD69-PE (341652, BD Biosciences) with or without CD14-APC-Cy7 (333951; BD Biosciences) for 30 minutes at 4°C followed by washing and acquisition on a five laser BD LSR Fortessa and analysis using FlowJo. In assays of NK cell–mediated cytotoxicity against PANC-1 cells, enriched NK cells were stimulated overnight with IL2 in low-adherence plates in the presence or absence of healthy donor–derived monocytes with or without 100 µmol/L HDC, 200 U/mL catalase (Sigma-Aldrich), 10 µmol/L GSK2795039 (MedChemExpress) or 1% DMSO (Sigma-Aldrich) followed by coculturing with carboxyfluorescein diacetate succinimidyl ester (Thermo Fisher Scientific) stained PANC-1 cells and anti-CD107a-BUV395 (BD Biosciences) for 4 hours at 37°C and 5% CO_2_. The NK cell:PANC-1 cell:monocyte ratio was 1:1:0.5. After incubation, the cells were prepared for flow cytometry as described above.

### Detection of Extracellular ROS

Superoxide anion production was measured by isoluminol-enhanced chemiluminescence (CL) that captures only extracellular ROS (28). In brief, healthy donor or patient-derived postoperative CD33^+^ cells were diluted to 0.5 million cells/mL in Krebs-Ringer glucose buffer supplemented with isoluminol (10 µg/mL; Sigma-Aldrich) and horseradish peroxidase (4 U/mL; Merck) and incubated in 96-well plates that had been coated or not with 10 mg/mL rituximab (Roche) and HDC (100 µmol/L) at 37°C. The release of extracellular ROS (light emission) was continuously monitored for 200 minutes using BMG FLUOStar Microplate Reader (BMG Labtech).

### Culture of B16F10 Cells

B16F10 cells were obtained in 2013 from the Cell Culture Laboratory at the Department of Virology, University of Gothenburg (Gothenburg, Sweden). The B16F10 genetic profile was analyzed by PCR (Idexx Bioanalytics) in 2018 and showed only minor genetic changes (addition of an allele at marker MCA-5-5) compared with the original cell line. Absence of *Mycoplasma* was confirmed using PCR (Eurofins Genomics). Aliquots of B16F10 cells were thawed and cultured in IMDM supplemented with 10% heat-inactivated FCS, 100 µg/mL penicillin, 100 µg/mL streptomycin, 1 mmol/L sodium pyruvate, and 2 mmol/L l-glutamine at 37°C and 5% CO_2_ for no more than 1 week prior to each *in vivo* experiment.

### Model of NK Cell–dependent Metastasis

All animal experiments were performed at the Experimental Biomedicine animal facility at Gothenburg University at sterile conditions with unlimited supply of food and water. Six to 8 weeks old female C57BL6/J (wild-type; WT) mice were obtained from Charles River Laboratories and underwent a 1-week acclimatization period prior to being used in experiments. B6.129S6-Cybbtm1din (*Nox2* knockout; KO) mice were obtained from Jackson Laboratories and bred in-house under sterile conditions. Male and female *Nox2*-KO mice were 6–12 weeks old. All animal experiments were performed according to the institutional guidelines and approved by the Research Animal Ethics Committee in Gothenburg (applications 1696/18 and 2483/19).

For each experiment, 4–5 age- and sex-matched WT mice and 4–5 age- and sex-matched *Nox2*-KO mice were randomly assigned to control, sponge-bearing (inflammation) and/or treatment groups. Mice were ear tagged and animals from different experimental groups were housed in the same cages. Treatment and tumor cell inoculation were performed in random orders between groups. In initial experiments only groups of control and sponge-bearing WT mice were included to define the effect of inflammation on M-MDSC and metastasis. Samples size was based on the magnitude of the inflammatory effect on metastasis.

PVA sponges (V.A.C. white foam dressing, KCI) were cut into 4 × 4 × 2 mm pieces and soaked in 70% EtOH in a 6-well plate for 1 minute, transferred to PBS and kept in PBS until use. Mice were anesthetized using 2.5% isoflurane, shaved on the back and wiped with iodine solution followed by 70% EtOH. A 1 cm incision was made 1 cm above the left hip joint and using blunt-end forceps, a pocket was created underneath the skin in which the sponge was inserted. The wound was closed with 7 mm clips (Agnthos). HDC-treated mice received 1.5 mg HDC in 100 µL physiologic NaCl by intraperitoneal injection every other day starting 2 days before sponge implantation and until day 11 thereafter. At 7 days after sponge implantation, blood samples were collected from vena saphena for analysis by flow cytometry. The following day mice were intravenously inoculated with 100,000 (for WT mice) or 150,000 (for KO mice) B16F10 melanoma cells. After 19–21 days, mice were sacrificed and pulmonary metastases were enumerated under a light microscope by an unbiased observer that was unaware of treatment group allocations.

### Immunophenotyping of Mouse Blood

Mouse blood was collected 1 week after sponge implantation from vena saphena in Ethylenediaminetetraacetic acid (EDTA) tubes. Red blood cells were lysed after which cells were stained with a myeloid panel of antibodies comprising Ly6G-FITC (561105, BD Biosciences), Ly6C-BV605 (563011, BD Biosciences), and CD11b-BV711 (563168, BD Biosciences) for 30 minutes at 4°C. Cells were washed and resuspended in DAPI (Invitrogen) before acquisition on a five laser BD LSR Fortessa and analyzed with DIVA (BD Biosciences).

To determine intracellular ROS levels, cells were stained with Ly6C-BV605 (563011, BD Biosciences) and CD11b-BV711 (563168, BD Biosciences) for 30 minutes at 4°C. After washing, cells were stained with H2-DCFDA and analyzed by flow cytometry as described above.

### Statistical Analysis

Statistical analyses were performed using GraphPad Prism (version 9 or later) and SPSS (version 28 or later) with figures prepared in GraphPad Prism. The mixed-effects analysis followed by Holm-Šídák multiple comparison test was used to compared preoperative and postoperative samples from patients and in assays of monocyte-induced NK cell suppression. Samples from patients and healthy donors were compared by the Kruskal–Wallis test followed by Dunn multiple comparison test.

Overall survival of patients was the time in days from day of surgery to death from any cause using data available on September 12, 2022, that is, when all patients had reached >32 months follow-up. Impact of immune-related and clinical parameters [sex, age, pathologic diagnosis including tumor stage, lymph node stage, American Society of Anaesthesiologists (ASA) physical status classification, presence of vein resection, duration of surgery, surgery-associated complications, and type of chemotherapy] on patient survival was analyzed by log-rank statistics and with Cox regression. For log-rank analysis of continuous variables patients were dichotomized using the Youden index. Parameters that reached a *P*-value below 0.15 in univariable Cox regression were included in multivariable Cox regression analysis. Linear regression was utilized for correlation analysis.

For expression analysis in a larger cohort of patients, we utilized the Kaplan–Meier plotter database (kmplot.com) that includes transcriptome data from 852 PDAC samples along with patient survival data. Impact of expression of selected genes on survival was analyzed using the best cut-off feature of the portal. For consistency, this portal was also used for survival analysis based on intratumoral gene expression within the IPEP cohort using the custom option. Kaplan–Meier survival plots with HR and log-rank *P* values were obtained in the portal (29, 30).

Single-cell PDAC transcriptome data from the Single Cell portal (singlecell.broadinstitute.org; 50516 cells, human treated PDAC single nucleus RNA-sequencing (sNuc-seq) was used to define the identity of cells expressing selected genes.

One-way ANOVA followed by Holm-Šídák multiple comparison test was used to define antimetastatic effects of HDC in mouse experiments. *P* values (two-sided) were designated as follows: *^/#^, *P* < 0.05; **^/##^, *P* < 0.01; ***^/###^, *P* < 0.001; and ****^/####^, *P* < 0.0001.

### Data Availability

Deidentified data on immunophenotyping and clinical outcome for the patients with pancreatic cancer may be available upon request to the corresponding author at anna.martner@gu.se.

## Results

### Systemic Inflammation and Lymphocyte Reduction after Pancreatic Cancer Surgery

This study included 17 patients with periampullary cancer who underwent major pancreatic cancer surgery with curative intent. Table 1 accounts for characteristics of patients and treatments along with their potential impact on survival.

Preoperative and postoperative whole blood, PBMC, and plasma samples were collected before surgery and 1, 3–5, and 28 days thereafter (Fig. 1B). Differential counts of whole blood showed that surgery entailed accumulation of circulating neutrophils with a trend toward increased monocyte counts (Fig. 1C and D). Counts of circulating lymphocytes were reduced in the postoperative phase (Fig. 1E). Analysis of multiple cytokines in plasma revealed pronounced induction of IL6 in the postsurgery week (Fig. 1F). Additional aspects of immune phenotypes and cytokines before and after surgery, including effects on eosinophils and levels of eotaxin, IL4 and GMCSF, are accounted for in Supplementary Fig. S1.

### MDSC with Enhanced Immunosuppressive Features Accumulate After Surgery

Compared with healthy donors, patients tended to have an increased proportion of cells with CD14^+^HLA-DR^low^ M-MDSC phenotype prior to surgery. The surgery entailed stark increase in absolute counts and frequencies of M-MDSC in the postoperative week with levels returning to preoperative levels after 4 weeks (Fig. 1G–I). Preoperative levels of intracellular ROS in M-MDSC were higher in patients than in healthy controls. ROS levels in patient M-MDSC further increased during the first week after surgery (Fig. 1J). Preoperative M-MDSC expressed high levels of the immunosuppressive ligand PD-L1 that further increased after surgery. Surgery additionally triggered augmented expression of HLA-ABC and reduced expression of the costimulatory molecule CD86 on M-MDSC (Supplementary Fig. S2).

### Compromised NK Cell Populations in Blood after Surgery

**FIGURE 2:**
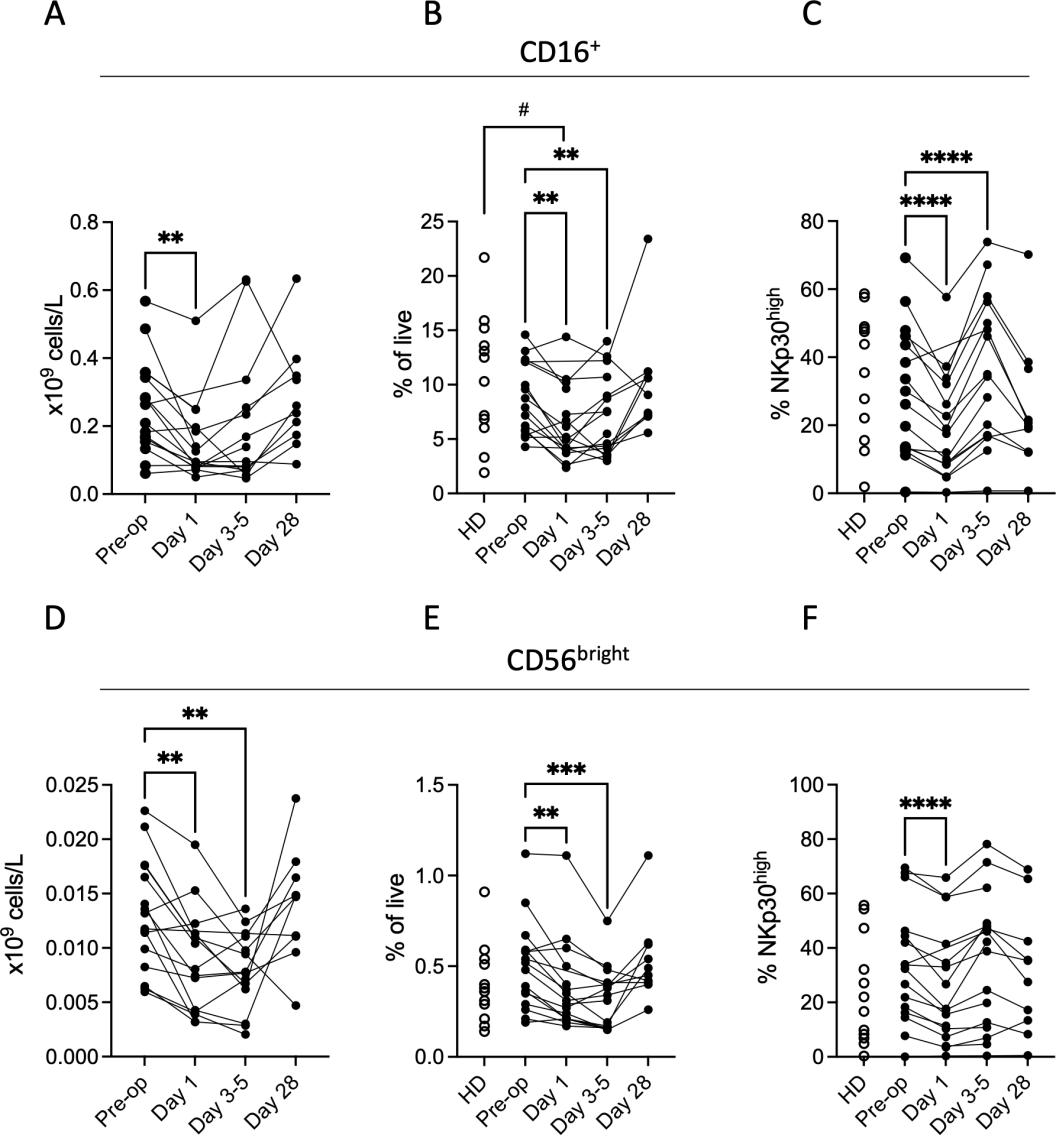
Pancreatic cancer surgery entails reduced levels of NK cells with abridged expression of the activating NK cell receptor NKp30. Absolute numbers (**A**, **D**) and frequencies (**B**, **E**) of CD16^+^ (**A**, **B**) and CD56^bright^ (**D**, **E**) NK cells in preoperative (pre-op) and postoperative (day 1, days 3–5, and day 28) samples from patients with periampullary cancer and healthy donors (HD). Frequencies of NKp30^high^-expressing cells among CD16^+^ (**C**) and CD56^bright^ (**F**) NK cells (*N* = 16 for pre-op, *N* = 15 for day 1, *N* = 14 days 3–5, and *N* = 10 for day 28 for A and D and *N* = 16 for pre-op, day 1 and days 3–5, *N* = 10 for day 28, *N* = 10 for HD for B–C and E–F). Preoperative and postoperative samples were compared using mixed-effects analysis followed by Šídák multiple comparison test (*P*-values indicated by *). HD and cancer samples were compared using Kruskal–Wallis test followed by Dunn multiple comparison test (*P*-values indicated by ^#^). ^#^, *P* < 0.05; **, *P* < 0.01; ***, *P* < 0.001; ****, *P* < 0.0001.

Human NK cells comprise two main subsets, the cytotoxic CD16^+^CD56^dim^ (CD16^+^) cells and the cytokine-producing CD56^bright^ cells (31, 32). Surgery-induced accumulation of M-MDSC coincided with reduced counts and frequencies of CD16^+^ NK cells and CD56^bright^ NK cells (Fig. 2). NK cells express activating receptors, including NKG2D and DNAM1 and the natural cytotoxicity receptors NKp30 and NKp46 (31). Shortly after surgery, the proportion of NK cells with high expression of NKp30 was reduced with a rebound effect noted at 3–5 days (Fig. 2C and F). Surgery also entailed reduced median fluorescence intensity (MFI) expression of NKp30, NKG2D, and DNAM1 on CD16^+^ NK cells, and of NKp30, NKp46 and DNAM1 on CD56^bright^ NK cells (Supplementary Fig. S3). Furthermore, surgery was associated with reduced frequencies of CD56^bright^ NK cells expressing NKG2A, an NK cell inhibitory receptor (32), and increased frequencies of CD56^bright^ NK cells expressing CD57, an NK cell maturation marker (ref. 32; Supplementary Fig. S3).

The surgery was additionally accompanied by reduced absolute counts and frequencies of CD4^+^ T cells and regulatory T cells along with reduced counts of CD8^+^ T cells (Supplementary Fig. S4). CD4^+^ T cells and regulatory T cells in patients showed increased expression of PD-1 before surgery. PD-1 expression was further increased on regulatory T cells and CD8^+^ T cells in the postoperative phase (Supplementary Fig. S4).

### Surgery Reduces Counts of Dendritic Cells

The counts and frequencies of antigen-presenting dendritic cells (DC) in blood were reduced after surgery (Supplementary Fig. S5A and S5B) along with reduced expression of the costimulatory molecules CD86 and HLA-DR on DC (Supplementary Fig. S5C and S5D). Surgery also reduced PD-L1 expression on DC (Supplementary Fig. S5E). The expression of HLA-ABC on DC was higher in patients than in healthy donors (Supplementary Fig. S5F).

### High Levels of M-MDSC Herald Poor Survival

**FIGURE 3:**
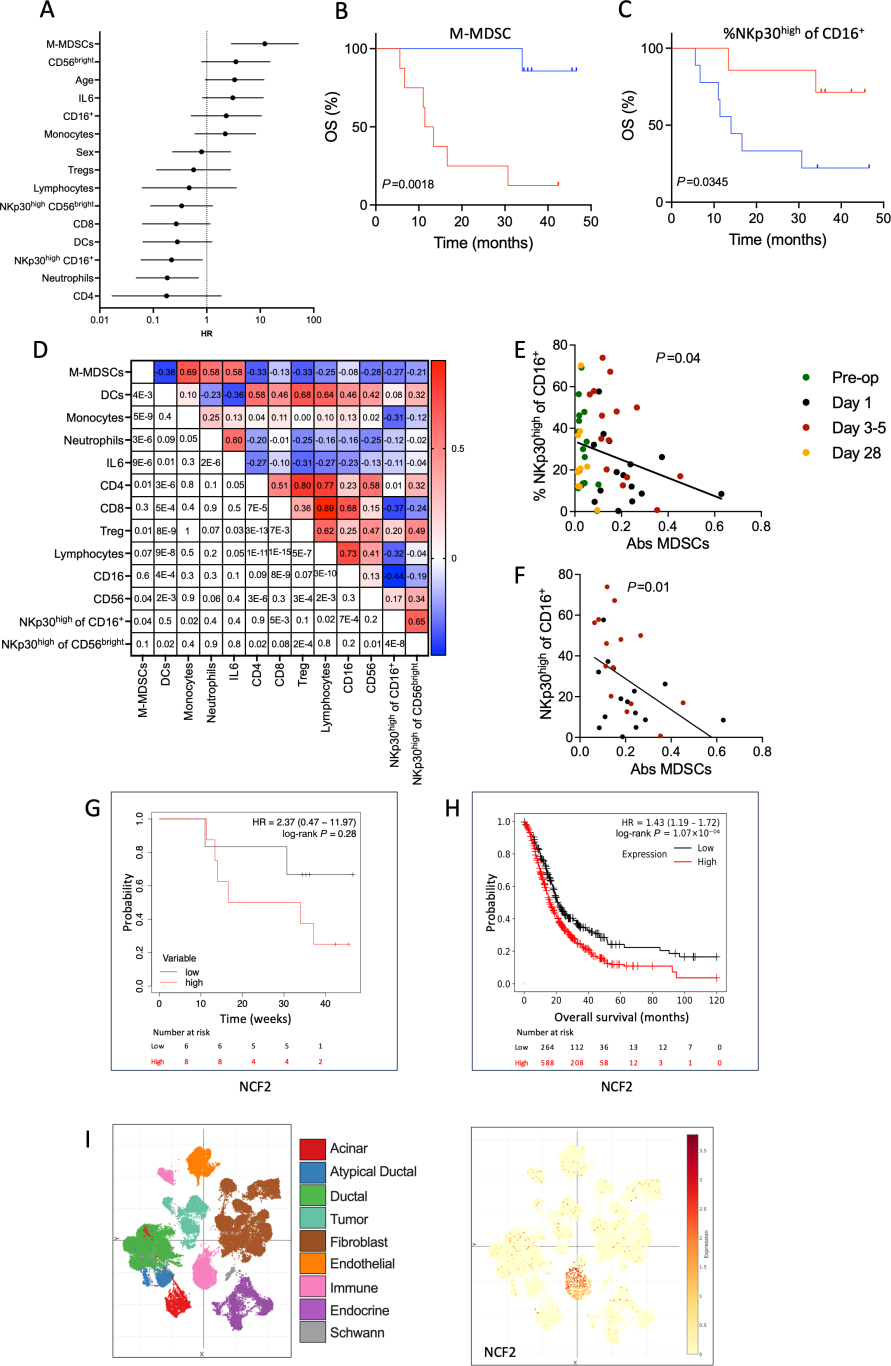
High levels of M-MDSC and low levels of NKp30^high^ NK cells after pancreatic cancer surgery correlate and predict poor survival. **A,** Forest plots for postoperative survival were generated by log-rank tests where patients were dichotomized on the basis of Youden index for high or low absolute number of M-MDSCs, CD56^bright^ NK cells, CD16^+^ NK cells, monocytes, lymphocytes, CD8^+^ T cells, regulatory T cells, CD4^+^ T cells, DC, neutrophils, or plasma levels of IL6, high expression level of NKp30^+^ cells among CD16^+^ (NKp30^high^ CD16^+^) and CD56^bright^ (NKp30^high^ CD56^bright^) NK cells one day after surgery or by age or sex. Kaplan–Meier survival comparisons by high (red) or low (blue) absolute numbers of M-MDSC (**B**) and NKp30^high^ cells among CD16^+^ NK cells (**C**) one day after surgery by log-rank test. **D,** Heat map of correlations (Pearson *r*, above the diagonal) between immune cell populations and IL6 among patients undergoing pancreatic cancer surgery. *P*-values corresponding to each correlation are shown below the diagonal. Correlation between absolute numbers of M-MDSC and frequencies of NKp30^high^ cells among CD16^+^ NK cells at all analyzed timepoints (**E**), or within the first week after surgery (green dots: pre-op, black dots: 1 day, red dots 3–5 days and orange dots 28 days after surgery; **F**). Statistics by linear regression. Survival impact of high (red) or low (black) intratumoral NCF2 expression in pancreas cancer samples from the IPEP study (*n* = 14; **G**) or the Kaplan–Meier plotter database (*n* = 852; **H**), using the best cut-off feature of the portal and log-rank statistics. **I,** Expression analysis of Single Cell portal PDAC samples showed that the majority of NCF2-expressing cells were confined to a specific immune cell cluster.

The surgery-induced skewing of immune phenotypes was pronounced early in the postoperative phase. To explore which phenotypes and cytokines altered by surgery that may impact on long-term survival, patients were dichotomized by Youden index based on having high or low numbers of immune cell phenotypes or plasma levels of IL6 the day after surgery followed by Kaplan–Meier statistics of survival. The most prominent effect of surgery on immunity was the induction of M-MDSC in the early postoperative phase. Patients with high absolute counts of M-MDSC one day after pancreas surgery showed poor long-term survival (*P* = 0.002, log-rank test, Fig. 3A and B). This association remained significant in multivariable Cox regression analysis taking age, sex, tumor stage, lymph node stage, ASA classification, presence of vein resection, complications during surgery, duration of surgery, and type of chemotherapy into account (Table 2). Subgroup analysis of patients with only PDAC (*n* = 14) showed that high levels of M-MDSC the day after surgery predicted poor survival (*P* = 0.01, log-rank test; *P* = 0.02 Cox regression) also in this group. High levels of M-MDSC at 3–5 days after surgery associated significantly with poor survival, with a similar trend for presurgery M-MDSC levels (Table 2).

**TABLE 2:** Cox regression analysis of absolute numbers of M-MDSC and frequency of NKp30^high^ of CD16^+^ NK cells on overall survival in patients with periampullary cancer before (preoperative) and at different days after (days 1, 3–5, 28) surgery. Covariates with impact on overall survival in univariable analysis (*P*-values <0.15) were included in multivariable analysis

Cell population (day)	Univariable analysis *P*-value	Multivariable analysis^a^ *P*-value
M-MDSCs (preoperative)	0.048	0.209
M-MDSCs (day 1)	0.006	0.019
M-MDSCs (days 3–5)	0.013	0.034
M-MDSCs (day 28)	0.763	—
NKp30^high^ of CD16^+^ NK cells (preoperative)	0.095	0.114
NKp30^high^ of CD16^+^ NK cells (day 1)	0.144	0.152
NKp30^high^ of CD16^+^ NK cells (days 3–5)	0.083	0.092
NKp30^high^ of CD16^+^ NK cells (day 28)	0.123	0.151

^a^Covariates considered: age, sex, tumor stage, lymph node stage, ASA classification, presence of vein resection, surgery duration, type of surgery, grade of complication, and type of chemotherapy.

In contrast, high frequencies of NKp30^high^ cells among CD16^+^ NK cells on the day after surgery significantly associated with improved overall survival (*P* = 0.03, Fig. 3A and C). High frequencies of NKp30^high^ CD16^+^ NK cells similarly prognosticated favorable survival when analyzed before surgery and on days 3–5 or 28 (*P* = 0.008, *P* = 0.01, and *P* = 0.001, respectively, log-rank test) with similar albeit nonsignificant trends in Cox regression analysis (Table 2). In accordance, high median expression of NKp30 on CD16^+^ NK cells associated with better survival at all timepoints (preoperative: *P* = 0.0081, 1 day after surgery: *P* = 0.035, 3–5 days after surgery: *P* = 0.01, and 28 days after surgery: *P* = 0.01, log-rank test). The expression of other natural cytotoxicity receptors (NCR) did not significantly associate with postoperative survival.

High postoperative counts of CD8^+^ T cells tended to associate with favorable outcome, and high counts of CD4^+^ T cells on day 1 after surgery heralded significantly improved overall survival (Fig. 3A) although this trend did not remain significant in Cox regression analysis (*P* > 0.2) or on days 3–5 postsurgery. Unexpectedly, high neutrophil counts one day after surgery associated with favorable survival outcome in Kaplan–Meier analysis (Fig. 3A). However, this trend was not observed in Cox regression analysis (*P* > 0.5) and the opposite effect of neutrophil counts on survival was observed 3–5 days after surgery (*P* = 0.02, log-rank test).

### Negative Correlations Between Myeloid Cells and Lymphocyte Phenotypes in Blood

Figure 3D is a heat map of multiple correlations between immune phenotypes and mediators. M-MDSC were positively correlated with monocytes, neutrophils, and with IL6, a trigger of MDSC expansion and activation (ref. 33; Fig. 3D). M-MDSC levels correlated negatively with T-cell and NK cell populations as well as with the frequency of NKp30^high^ cells among CD16^+^ NK cells (Fig. 3D–F). As surgery altered several immune parameters in the postoperative week, we performed regression analyses with only these timepoints included. In the early postoperative period, there was a significant negative correlation between M-MDSC and frequency of NKp30^high^-expressing CD16^+^ NK cells (Fig. 3F), but not with other lymphocyte subsets.

### Intratumoral NOX2 Expression Predicts Poor Survival

Tumor biopsies obtained during surgery were analyzed for expression of *NCF2*, *CYBB, CD68*, *CD163,* and *NCAM1* by RT-PCR. No significant correlations were noted between intratumoral myeloid cell marker expression and systemic myeloid cell levels at baseline. However, intratumoral expression of the NOX2 subunits *NCF2* and *CYBB* correlated with DCFDA levels in systemic CD33^+^ myeloid cells (*P* = 0.007 and *P* = 0.018, respectively, *n* = 14). In survival analysis, high intratumoral expression of NOX2 and myeloid cell markers tended to negatively impact on survival, with an opposite trend for *NCAM1*, encoding the NK cell marker CD56 (Fig. 3G; Supplementary Fig. S6).

Transcriptome data from 852 PDAC patient samples available in the Kaplan–Meier plotter database (kmplot.com) were analyzed to investigate survival impact of selected genes in a larger patient cohort. High expression of the NOX2 subunits *NCF2*, *CYBB* and the M2-type macrophage marker *CD163*, but not the pan-macrophage marker *CD68*, significantly associated with shorter overall survival (Fig. 3H; Supplementary Fig. S6E–S6G). Analysis of PDAC single-cell expression data, available in the Single Cell portal (singlecell.broadinstitute.org), showed that these markers were expressed within the same immune cell subset, as exemplified by *NCF2* and *CD163* (Fig. 3I; Supplementary Fig. S6L).

The Kaplan–Meier plotter was also used to investigate survival impact of cytotoxic lymphocyte markers in PDAC. Intratumoral expression of several NK and T-cell markers, including *PRF1*, *CD226* (encoding DNAM-1)*, NCAM1* and *CD8A*, associated with longer survival (Supplementary Fig. S6H–S6K). PDAC single-cell expression data showed that these markers were mainly expressed by a separate cluster of immune cells, as exemplified by *PRF1*, encoding perforin-1 expressed by NK cells and cytotoxic T cells (Supplementary Fig. S6M).

### Inhibition of NOX2-derived ROS from Myeloid Cells Restores NK Cell Killing of Human Pancreatic Cancer Cells

**FIGURE 4:**
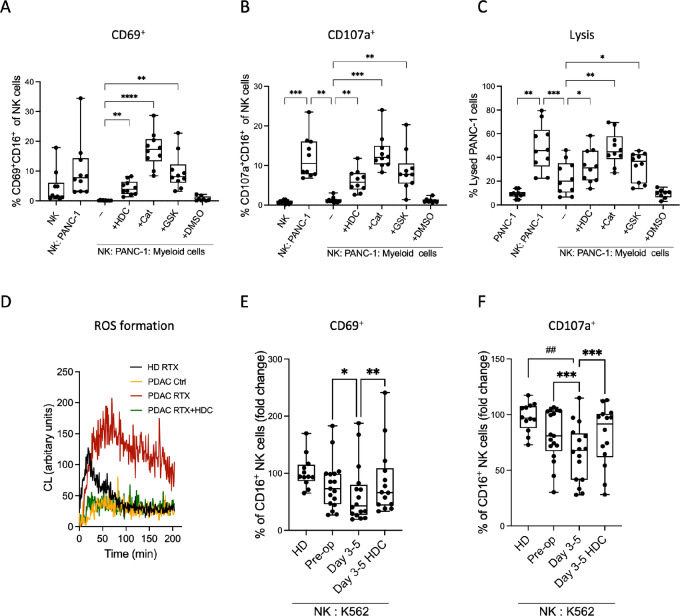
M-MDSC suppress NK cells by generating ROS and show enhanced NK cell suppression after surgery. **A–C,** NK cells were co-cultured with or without PANC-1 cells and freshly isolated healthy donor (HD) monocytes in the presence or absence of 100 µmol/L HDC, 200 U/mL catalase (Cat), 10 µmol/L GSK2795039 (GSK) or 1% DMSO (control). NK cell activation and degranulation were measured by flow cytometry and are shown by frequencies of NK cells expressing CD69^+^CD16^+^ (**A**) or CD107a^+^CD16^+^ (**B**), respectively. **C,** NK cell cytotoxicity against PANC-1 cells was determined by frequency of LIVE/DEAD stained PANC-1 cells. NK cell cytotoxicity against PANC-1 cells was measured in four independent experiments, that each included two to three combinations of NK cells and monocyte from different donors. **D,** ROS formation was measured by CL from CD33^+^ cells isolated 3–5 days after surgery in the absence of stimulation (PDAC Ctrl, orange), in the presence of rituximab (PDAC RTX, red), in the presence of rituximab and HDC (PDAC RTX + HDC, green) and from 2 HDs stimulated with rituximab (mean ROS formation shown, HD RTX, black). NK cells were cocultured with K562 cells and CD33^+^ cells isolated from HDs or patients before surgery (pre-op) or 3–5 days after surgery (days 3–5) in the presence (HDC) or absence of HDC. NK cell activation and degranulation were measured by flow cytometry as frequencies of CD16^+^ NK cells expressing CD69 (**E**) or CD107a (**F**) in NK:K562:Myeloid cell cocultures. NK cell cytotoxicity was measured in 10 independent experiments that were normalized by setting the mean NK cell cytotoxicity against K562 in the absence of myeloid cells to 1 in each experiment. Statistics by mixed-effects analysis followed by Šídák multiple comparison test (*P*-values indicated by *). HD and cancer samples were compared using Kruskal–Wallis test followed by Dunn multiple comparison test (*P*-values indicated by ^#^).*, *P* < 0.05; **^/^^##^, *P* < 0.01; ***, *P* < 0.001; ****, *P* < 0.0001.

These results identified two main immune perturbations induced by pancreatic surgery, that is, accumulation of myeloid cells, including M-MDSC, and concomitant reduction of NK cell number and function. We therefore asked whether myeloid cells may inhibit NK cell–mediated killing of pancreatic cancer cells. Human primary NK cells were cocultured with cells of the pancreatic cancer cell line PANC-1 as target cells in the presence or absence of primary monocytes. NK cells readily exerted cytotoxicity against PANC-1 cells as reflected by acquisition of the activation marker CD69 and the degranulation marker CD107a by the NK cells and by the expression of apoptosis markers by the pancreatic cancer cells at 4 hours of coculture (Fig. 4A–C). The presence of monocytes significantly prevented NK cell activation (Fig. 4A) as well as NK cell degranulation (Fig. 4B) and cytotoxicity (Fig. 4C) toward PANC-1 cells.

Myeloid cells have been shown to suppress adjacent NK and T cells by secreting NOX2-derived ROS (11, 34–36). To investigate whether myeloid cells may prevent NK cell–mediated killing of pancreatic cancer cells by this mechanism, the NOX2 inhibitor HDC (36, 37), the ROS scavenger catalase or the NOX2-specific inhibitor GSK2795039 (38) was added to cocultures of NK cells and primary myeloid cells. These ROS inhibitors significantly counteracted the observed immunosuppression and restored NK cell cytotoxicity against PANC-1 cells (Fig. 4A–C).

### Surgery-induced MDSC Suppress NK Cell Function by Generating NOX2-derived ROS

We next explored whether the immunosuppressive features of myeloid cells were altered following pancreatic cancer surgery. As shown in Fig. 1H, patient M-MDSC contained higher levels of ROS than did healthy donor myeloid cells, and ROS levels were further increased after surgery. In accordance, CD33^+^ myeloid cells isolated in the postoperative phase of pancreatic cancer surgery produced higher ROS levels, compared with healthy donor myeloid cells (Fig. 4D). Furthermore, the NOX2 inhibitor HDC almost completely blocked ROS formation in postoperative samples from patient myeloid cells (Fig. 4D).

In additional experiments, CD33^+^ myeloid cells, including M-MDSC, were isolated from frozen samples obtained from patients before and after surgery, or from healthy donors. These MDSC-like cells were added to cocultures of primary NK cells and the prototypic NK cell–sensitive target cells K562. NK cell function, as determined by acquisition the NK cell degranulation marker CD107a at 4 hours of coincubation with target cells, was slightly more suppressed by myeloid cells isolated from patients before surgery, compared with myeloid cells isolated from healthy donors [*P* = 0.048 *n* = 12 (healthy donors), *n* = 17 (preoperative samples), Mann–Whitney test]. Furthermore, myeloid cells isolated from postoperative patient samples were significantly more suppressive toward NK cell function than preoperative samples (Fig. 4E and F). Addition of the NOX2 inhibitor HDC to the cocultures significantly prevented the postoperative myeloid cell–induced suppression of NK cell function (Fig. 4E and F).

### Surgical Stress Activates NOX2 to Promote Experimental Metastasis

**FIGURE 5:**
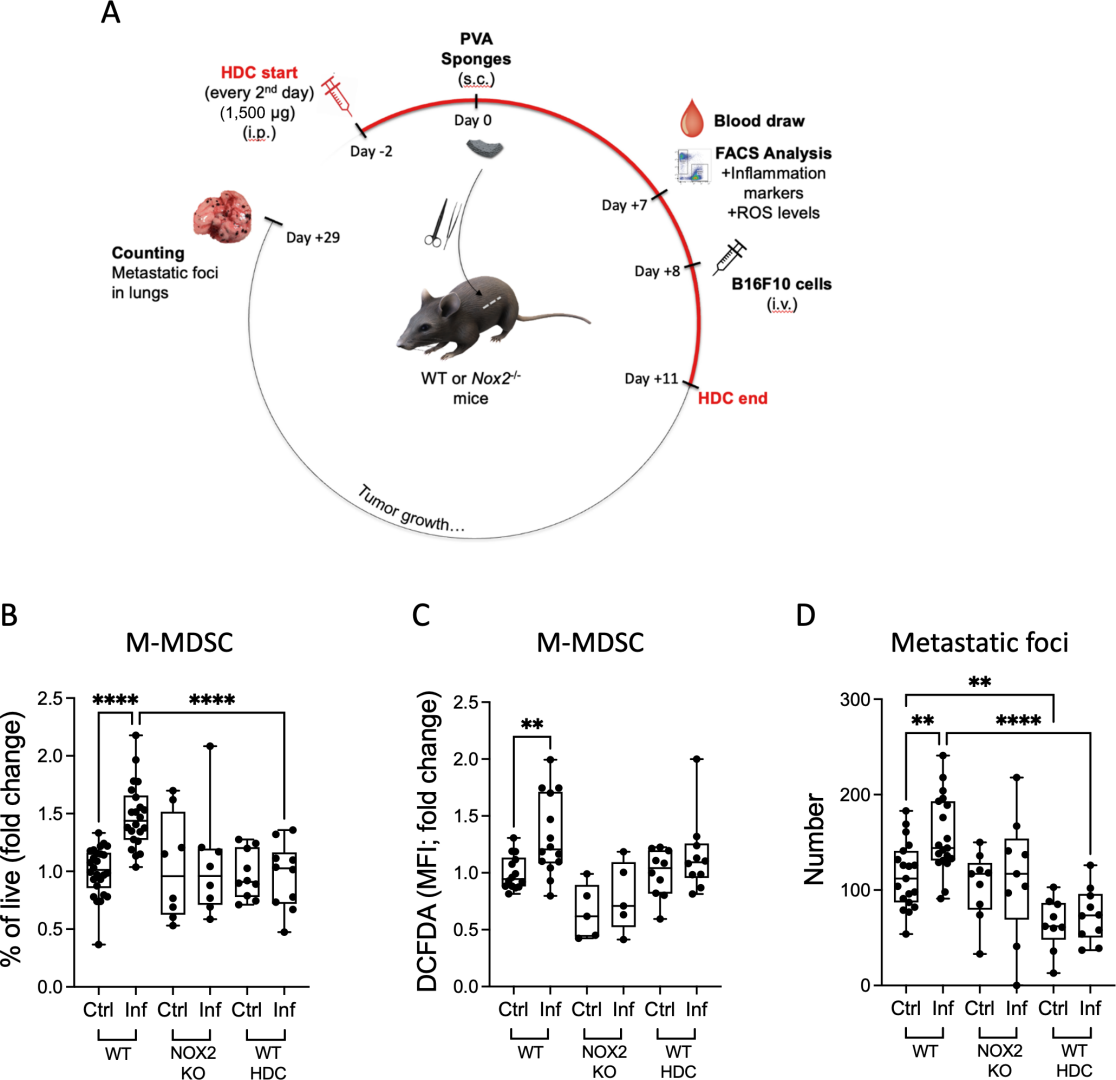
NOX2 inhibition prevents surgery-induced metastasis in a murine model. **A,** Schematic outline of the experimental model of surgery-induced metastasis. Blood samples were collected from naïve (Ctrl) and sponge-bearing (Inf) WT and NOX2-KO mice 1 week after a surgical procedure (sponge implantation). A group of WT mice received intraperitoneal injections with histamine dihydrochloride (WT HDC) every other day starting one day before surgery and continuing until 3 days after tumor cell challenge. Frequency of CD11b^+^Ly6C^+^M-MDSC (**B**) and intracellular ROS levels in M-MDSC (**C**) were measured by flow cytometry. At 8 days postsurgery, mice were intravenously inoculated with B16F10 cells. **D,** Metastatic lesions in lungs were enumerated 19–21 days after tumor cell challenge. Differences in inflammatory monocytes, DCFDA levels, and tumor numbers *in vivo* were calculated using one-way ANOVA following Holm-Šídák multiple comparisons test. The frequency of M-MDSC and number of metastatic foci was evaluated in four independent experiments for WT mice with or without surgery-induced inflammation, and in two experiments for KO mice and HDC treated mice. DCFDA levels of M-MDSC were measured in three independent experiments for WT mice, two experiments for KO mice, and one experiment for HDC-treated mice. In each experiment, 4–5 mice per group were included. Fold change was calculated by dividing raw data with the mean of raw data in the WT mice control group in each experiment. *, *P* < 0.05; **, *P* < 0.01; ***, *P* < 0.001; ****, *P* < 0.0001.

Subcutaneous implantation of sponges in mice has been reported to trigger surgical stress and systemic inflammation that promotes distant metastasis in a process largely driven by neutrophils, inflammatory (Ly6C^hi^) monocytes and their associated cytokines (27). We utilized this model of surgical stress to explore whether genetic or pharmacologic targeting of NOX2 prevents surgery-induced metastasis of B16F10 tumor cells. Hematogenous metastasis of B16F10 cells is highly controlled by NK cells (34, 39, 40). Sterile sponges were implanted subcutaneously to WT and *Nox2*-KO mice. Blood samples were analyzed for inflammatory markers 1 week later. The surgically stressed mice were then intravenously challenged with B16F10 tumor cells and macroscopic pulmonary metastases were enumerated 3 weeks later (Fig. 5A).

In mice, M-MDSC are phenotypically defined by the expression of CD11b and Ly6C (12). We observed pronounced expansion of cells with M-MDSC phenotype in surgically stressed WT mice, along with elevated levels of cellular ROS (Fig. 5B and C). In contrast, no increase in M-MDSC frequency or ROS levels was noted in surgically stressed *Nox2*-KO mice (Fig. 5B and C). *In vivo* administration of the NOX2 inhibitor HDC completely inhibited sponge-induced expansion of M-MDSC with a similar trend for ROS levels (Fig. 5B and C).

Surgically stressed WT mice showed increased metastasis compared with WT control mice. No increase of metastasis was observed in surgically stressed *Nox2*-KO mice (Fig. 5D). Pharmacologic NOX2 inhibition by HDC treatment *in vivo* markedly reduced metastasis in control mice and in surgically stressed WT mice, but not in *Nox2*-KO mice (Fig. 5D).

## Discussion

Human periampullary cancer, including PDAC, comprises an immunosuppressive milieu of infiltrating myeloid cells, including monocyte/macrophages and neutrophils, along with regulatory T cells, all of which are assumed to dampen antineoplastic immunity (17–19). Although mechanisms contributing to pancreatic cancer–related immunosuppression are likely multifaceted, a recurring theme is that infiltrating myeloid cells negatively impact on tumor cell clearance and on the effectiveness of immunotherapy (13, 14, 19, 27). This notion is bolstered by reports showing that the density of intratumoral or peritumoral neutrophils and macrophages are independent markers of advanced disease and poor survival in patients with metastatic pancreatic cancer (41, 42). Our analysis showing that high expression of *CD163*, which is expressed by M2-type macrophages, in resected pancreatic tumors associated with poor survival is coherent with these previous reports. Furthermore, we observed a negative survival impact of intratumoral expression of the NOX2 subunits *NCF2* and *CYBB*. Single-cell analysis showed that expression was largely confined to the myeloid immune cell subset. With the precaution that observed effect might be correlative rather than causative, the results suggest that myeloid cell ROS formation within the PDAC tumor microenvironment may negatively affect postoperative outcome.

The impact of myeloid cell function in blood on the survival of patients with cancer has mainly been studied by analysis of myeloid cell to lymphocyte ratios. A high neutrophil to lymphocyte ratio, predicts poor survival in metastatic pancreatic cancer with a similar, although less studied, negative survival impact of the monocyte to lymphocyte ratio (19, 23–25). Furthermore, preoperative inflammation-based scores, as well as restoration of neutrophil to lymphocyte ratio during neoadjuvant chemotherapy, were suggested to prognosticate postoperative survival also in patients with PDAC undergoing surgery with curative intent (23–25, 43, 44). To our knowledge, the prognostic impact of inflammation scores in the immediate postoperative stage was not previously studied. In our cohort, a high monocyte to lymphocyte ratio prior to surgery associated with poor survival (*P* = 0.035, *n* = 17), with a stronger impact the day after surgery (*P* = 0.0026, *n* = 16).

The main aim of our study was to define and determine the prognostic impact of surgery-induced systemic inflammation and immunosuppression. We focused the analysis on M-MDSC that are immature immunosuppressive monocytic cells that, in contrast to their granulocytic counterparts, withstand cryopreservation and thus are suitable for longitudinal analysis of stored clinical samples. It was observed that patients tended to harbor elevated counts of M-MDSC prior to surgery, but also that the surgical procedure entailed rapid and pronounced accumulation of these cells in blood. The accumulating M-MDSC expressed reduced levels of CD86 alongside enhanced levels of PD-L1 and MHC class I. While cytokines associated with IFN-related signaling were reduced in the postoperative phase, surgery triggered a pronounced increase of systemic IL6. This cytokine regulates accumulation and immunosuppressive features of MDSC, including PD-L1 expression (33), suggesting that induction of IL6 may have contributed to the postoperative accumulation of M-MDSC. Furthermore, postoperative levels of M-MDSC were predictive of poor survival with follow-up of >30 months. The negative impact of high postsurgical levels of M-MDSC on survival remained significant in multivariable analysis and similarly prognosticated survival within the subgroup of patients histopathologically diagnosed with PDAC.

Pancreatic cancer surgery is a prolonged and intricate procedure likely to give rise to systemic inflammation. The level of surgery-associated complications was within the expected range in this cohort (2/17 patients with a Clavien Dindo score of 3 or above) and the surgery-related complications did not significantly impact on survival (Table 1) or M-MDSC induction (*P* > 0.5 at both 1 and 3–5 days, *n* = 17, linear regression). One of the analyzed patients undergoing pancreaticoduodenectomy did not have cancer upon histopathologic examination of resected tissue. In this patient, surgery entailed a substantial rise in systemic M-MDSC with immunosuppressive features (Supplementary Fig. S7), suggesting that the surgery-induced immunosuppression is not specific to pancreatic cancer but rather to the extent of surgery. In accordance, previous studies report enhanced numbers and/or immunosuppressive features of MDSC following surgery in patients with lung cancer and rectal cancer (15, 45, 46). Also during critical illness, such as sepsis, immunosuppressive MDSC have been shown to accumulate as a result of emergency myelopoiesis (47).

We observed striking reduction of NK cell and T-cell counts in blood after surgery. NK cells also showed reduced expression of the NCR NKp30 in the early postoperative phase, which associated with poor survival. Similar albeit weaker trends were observed between postsurgical T-cell levels and survival. We performed correlative analyses to define the potential interplay between M-MDSC and antineoplastic lymphocytes following pancreatic cancer surgery and observed a negative correlation between accumulation of M-MDSC and presence of lymphocytes, including functional NKp30-expressing cytotoxic NK cells in blood. In the postoperative phase, M-MDSC expressed elevated DCFDA, a marker of ROS formation that may reflect the propensity of these cells to exert immunosuppression via the NOX2/ROS axis (37). The observed negative correlation between M-MDSC and NK cells after surgery is coherent with the earlier finding that human NK cells are highly susceptible to ROS-induced toxicity and functional inhibition (48).

We next performed *in vitro* and *ex vivo* experiments aiming to reconstitute M-MDSC–induced NK cell inhibition using MDSC-like cells recovered from healthy donors and patients with pancreatic cancer as suppressor cells in assays of NK cell functionality. M-MDSC, recovered in the early postoperative phase, were highly suppressive toward NK cells, which was reversed by pharmacologic NOX2 inhibition. Although our results do not exclude the participation by supplementary or alternative mechanisms of immunosuppression and immunosurveillance, we propose that the NOX2/ROS axis contributes to NK cell dysfunction in pancreatic cancer and that this pathway of immunosuppression may be relevant to the occurrence of metastasis. This view is bolstered by previous preclinical and clinical studies implying a role for NK cell function in control of distant metastasis (49–51). In further support of this assumption, mining of publicly available databases suggested that presence of cytotoxic lymphocytes, including NK cells, in PDAC tumors associated with prolonged survival.

In a final set of experiments, we utilized a previously reported murine model of NK cell–controlled metastasis (34, 39) and observed that a surgical procedure entailed aggravated distant metastasis, which was absent in *Nox2*-KO mice and in mice treated with a NOX2 inhibitor (HDC). A limitation is that non-pancreatic cancer target cells were utilized. This model thus aimed to define the role of surgical stress and NOX2 on NK cell–mediated clearance of malignant cells. The results are in line with studies showing that NK cell–mediated control of metastatic cells is compromised by NOX2-expressing myeloid cells (34, 52) and additionally imply that surgery-induced, NOX2-derived ROS dampen anti-metastatic functions of NK cells *in vivo*. Our findings also agree with the report by Krall and colleagues that a similar surgical procedure in mice carrying D2A1 mammary tumors entailed myeloid cell–induced inhibition of cytotoxic lymphocytes with ensuing aggravation of metastasis (27) and extend these earlier findings by implying a role for NOX2-derived ROS as conceivable mediators of surgery-induced immunosuppression.

We propose that strategies to target MDSC in the perioperative phase may limit the risk of metastatic spread after surgery. Several approaches, including targeting the CXCR2 pathway, the TRAIL pathway and the NOX2 pathway may be employed to reduce MDSC, as reviewed previously (53). The NOX2 inhibitor HDC is, in combination with low-dose IL2, approved for remission maintenance in acute myeloid leukemia in Europe (54). Postapproval studies imply that the reduced ROS formation from M-MDSC by the HDC component (55) and the induction and activation of NK cells by the IL2 component (56, 57) contribute to the anti-leukemic efficacy of this regimen. The results of the current study suggest that this HDC-based immunotherapy is conceivable also in solid malignancies for the prevention of metastasis in conditions of surgical stress.

This study has weaknesses, including a small study cohort. Also, the observed associations between M-MDSC and NK cell function, as well as the impact of these biomarkers on survival, may be correlative rather than causative. With these reservations our results identify NOX2-derived ROS produced by M-MDSC, and ensuing abridged NK cell functionality, as a potentially targetable mediator of immunosuppression in human periampullary cancer.

## Supplementary Material

Table S1 Sequences of primers used for RT-PCR

Figure S1 Levels of cytokines in plasma before and after pancreatic cancer surgery

Figure S2 Expression of CD86, HLA-ABC and PD-L1 on M-MDSC before and after surgery

Figure S3 Expression of NK cell receptors on NK cells before and after surgery

Figure S4 PD-1 expression, absolut counts and frequences of T cells in peripheral blood before and after surgery

Figure S5 DC frequences and DC marker expression before and after surgery

Figure S6 Impact of intratumoral expression of myeloid cell and lymphocyte markers on pancreatic cancer survival

Figure S7 M-MDSC induction in a non-cancerous patient undergoing pancreatic cancer surgery
