# Supplementary figures and images for: Impact of Surgery-Induced Myeloid-derived Suppressor Cells and the NOX2/ROS Axis on Postoperative Survival in Human Pancreatic Cancer
Source: Cancer Res Commun. 2024 Apr 25;4(4):1135–49. doi: 10.1158/2767-9764.CRC-23-0447 (PMC11044860; doi:10.1158/2767-9764.CRC-23-0447)

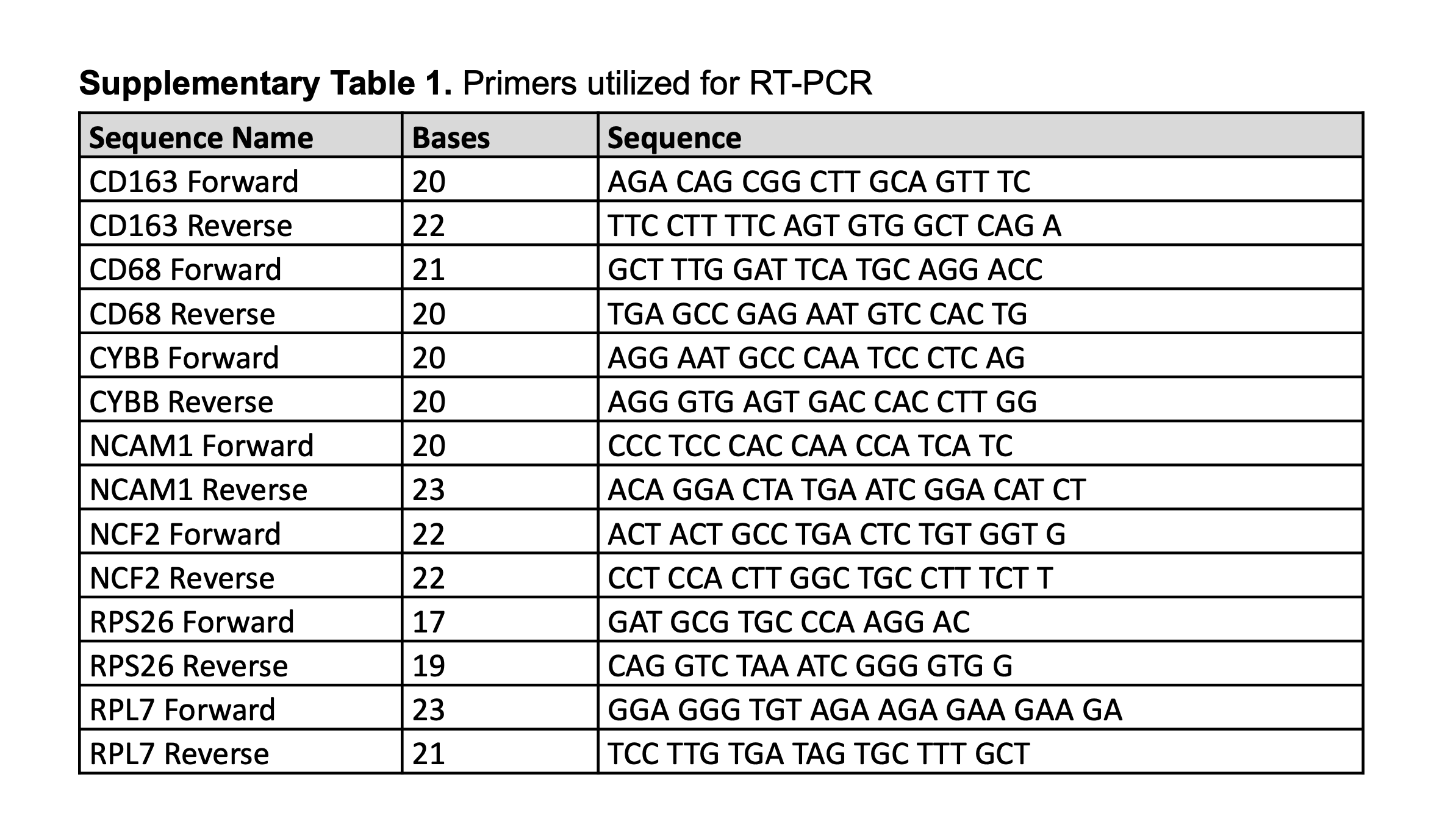

Supplement: Table S1 — Sequences of primers used for RT-PCR [file crc-23-0447-s01.png]

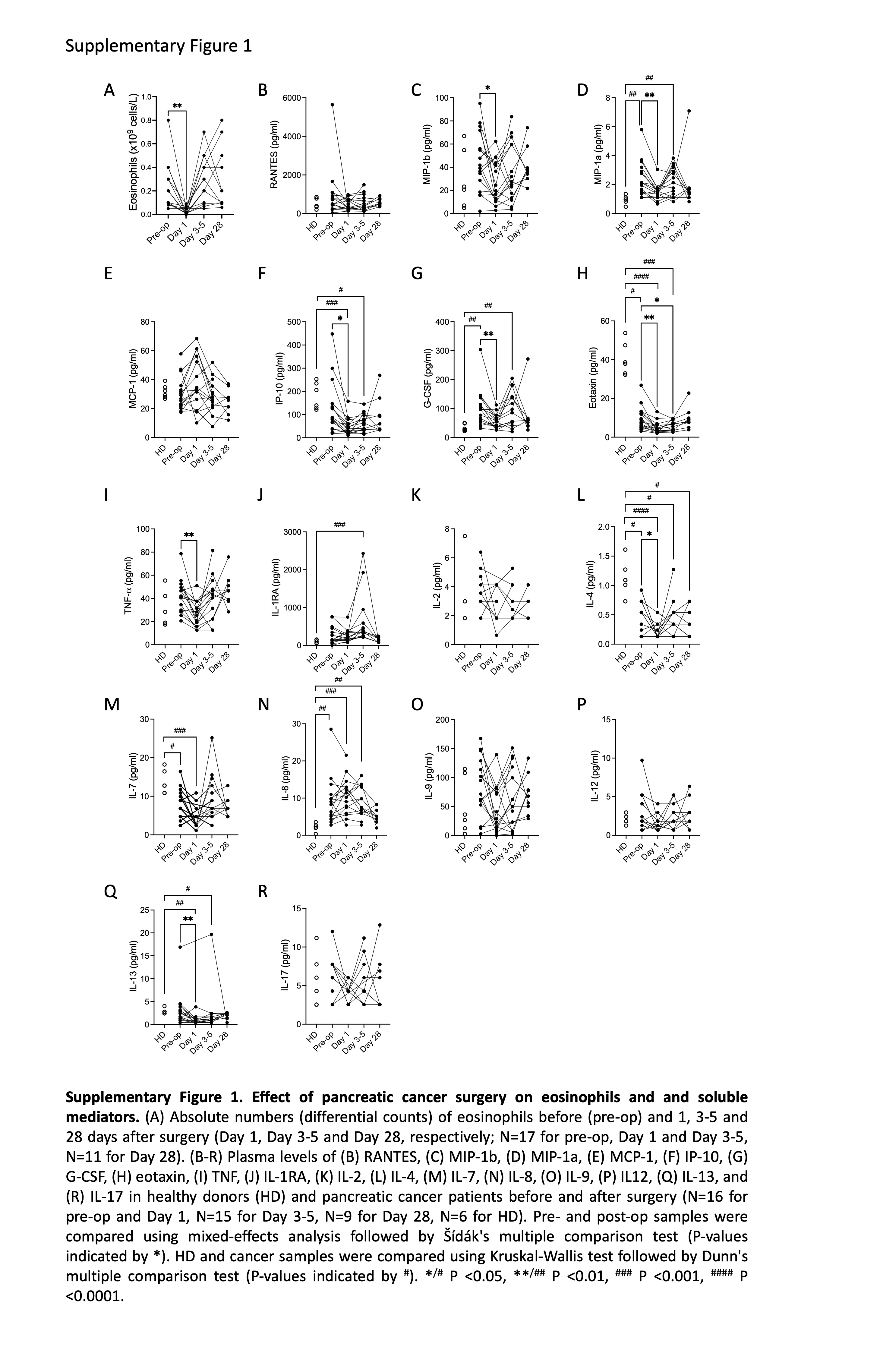

Supplement: Figure S1 — Levels of cytokines in plasma before and after pancreatic cancer surgery [file crc-23-0447-s02.png]

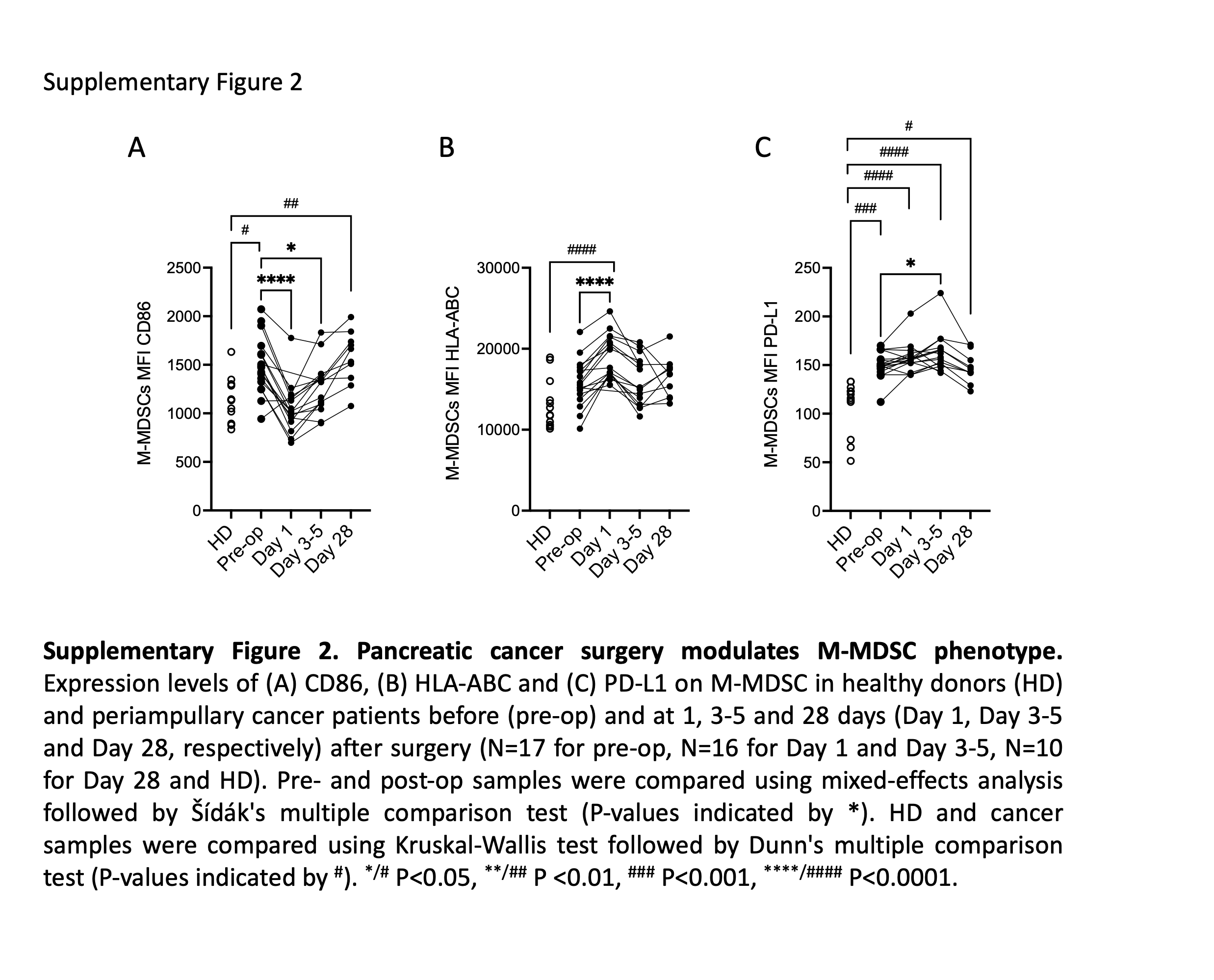

Supplement: Figure S2 — Expression of CD86, HLA-ABC and PD-L1 on M-MDSC before and after surgery [file crc-23-0447-s03.png]

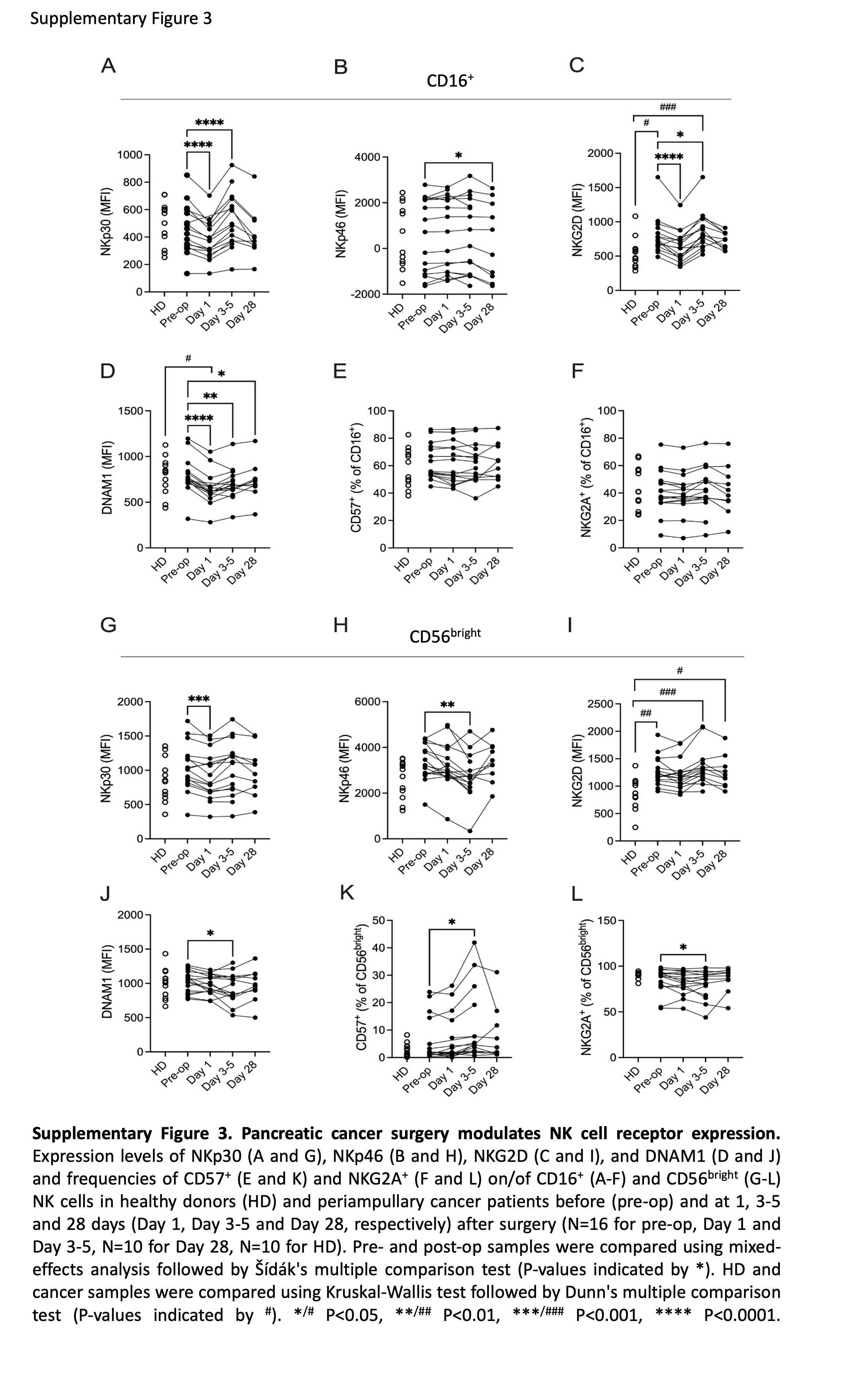

Supplement: Figure S3 — Expression of NK cell receptors on NK cells before and after surgery [file crc-23-0447-s04.png]

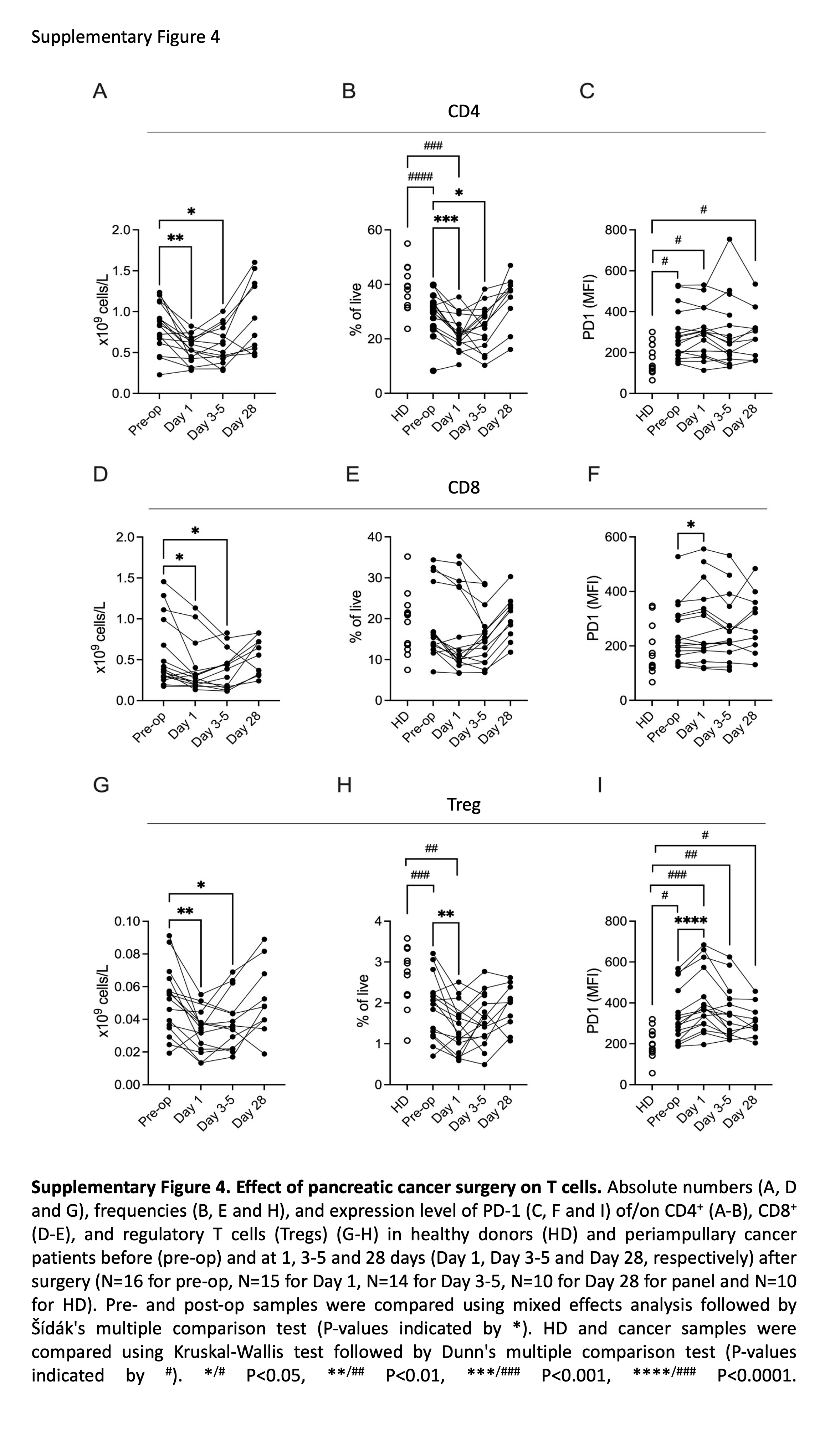

Supplement: Figure S4 — PD-1 expression, absolut counts and frequences of T cells in peripheral blood before and after surgery [file crc-23-0447-s05.png]

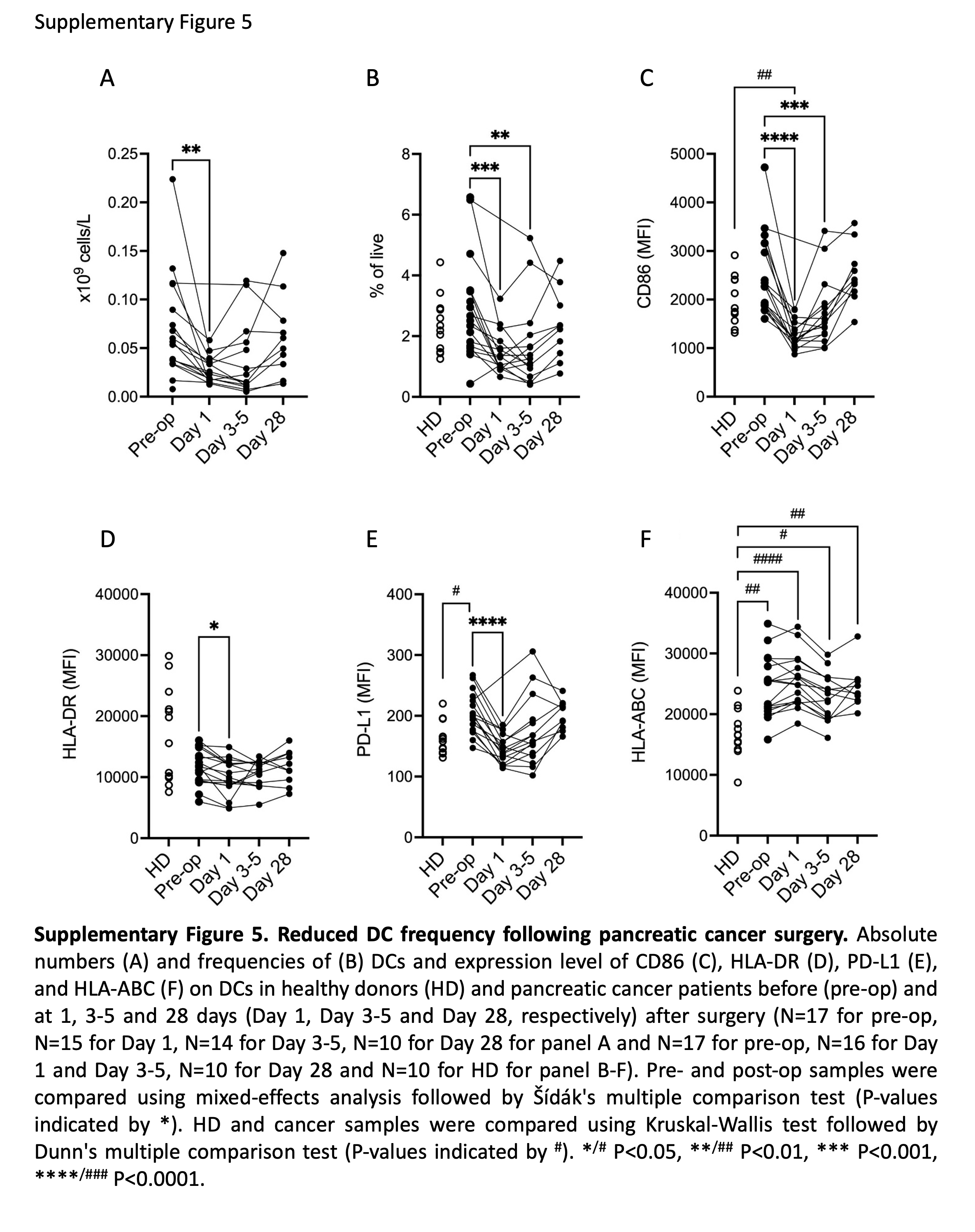

Supplement: Figure S5 — DC frequences and DC marker expression before and after surgery [file crc-23-0447-s06.png]

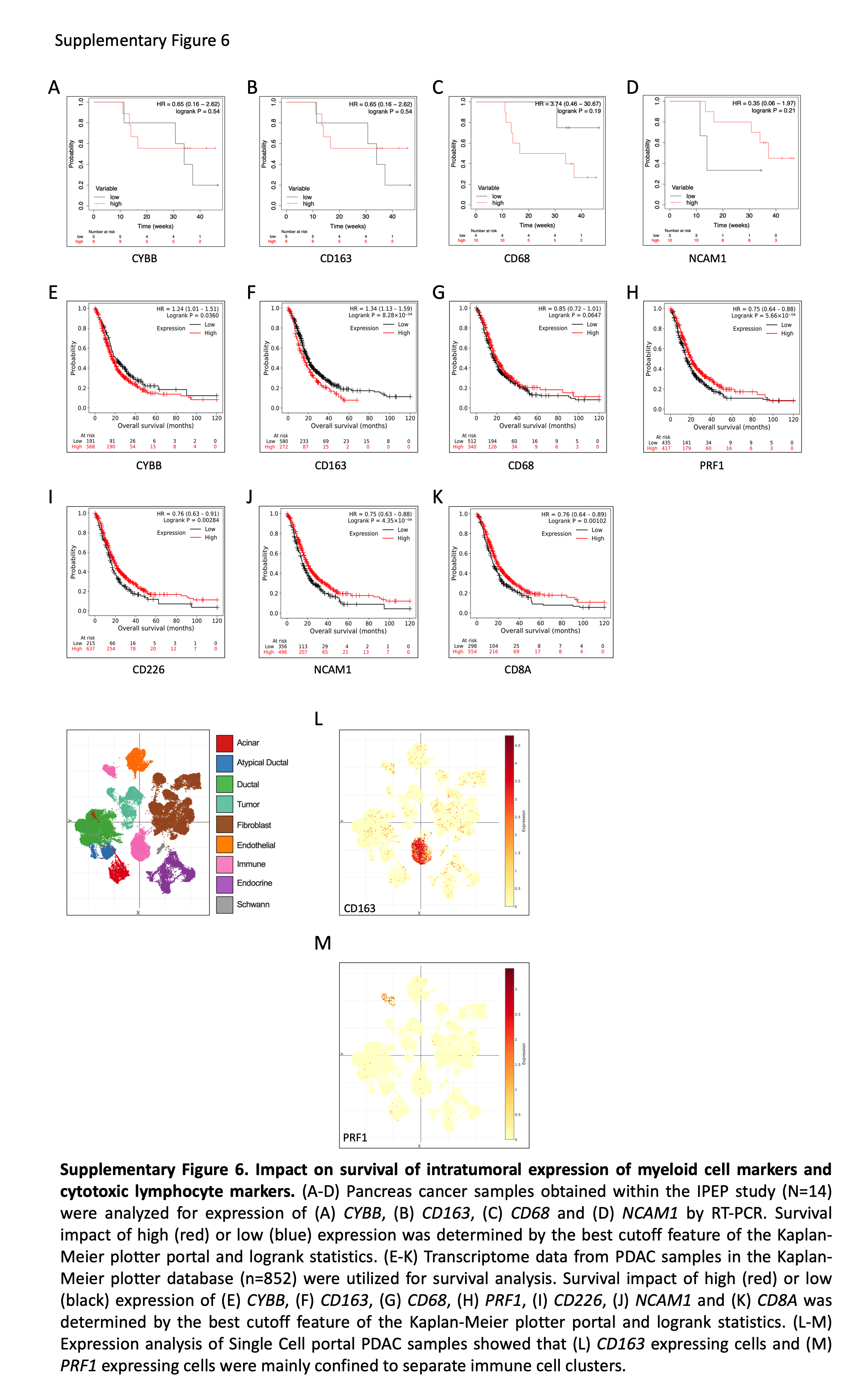

Supplement: Figure S6 — Impact of intratumoral expression of myeloid cell and lymphocyte markers on pancreatic cancer survival [file crc-23-0447-s07.png]

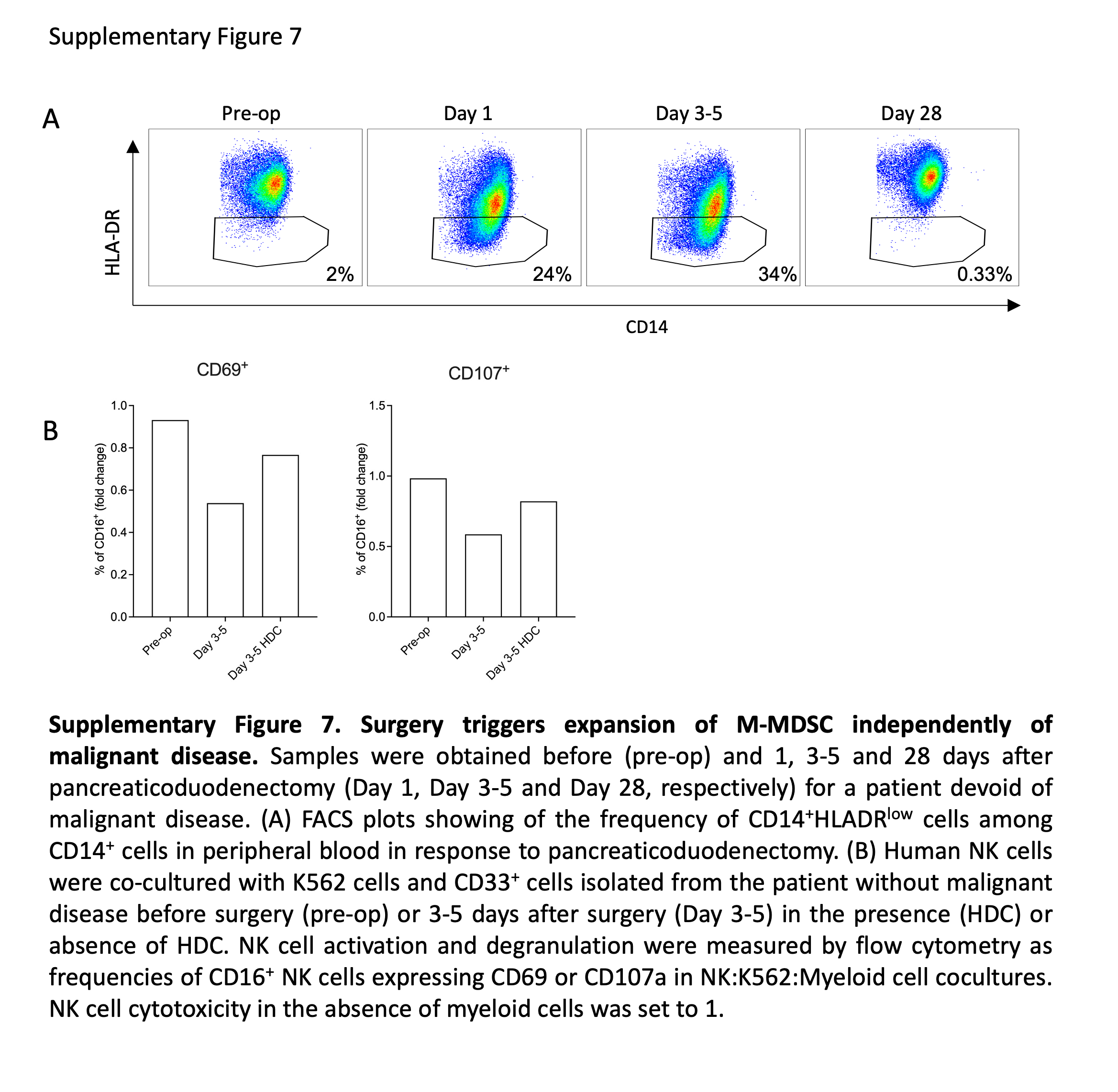

Supplement: Figure S7 — M-MDSC induction in a non-cancerous patient undergoing pancreatic cancer surgery [file crc-23-0447-s08.png]
